# CsBZIP40 confers resistance against citrus bacterial canker by repressing CsWRKY43-CsPrx53/CsSOD13 cascade mediated ROS scavenging

**DOI:** 10.1093/hr/uhad138

**Published:** 2023-07-11

**Authors:** Qiang Li, Xiujuan Qin, Miao Zhang, Qiyuan Yu, Ruirui Jia, Jie Fan, Xin Huang, Jia Fu, Chenxi Zhang, Baohang Xian, Wen Yang, Qin Long, Aihong Peng, Lixiao Yao, Shanchun Chen, Yongrui He

**Affiliations:** Citrus Research Institute, Southwest University/Chinese Academy of Agricultural Sciences, Beibei, Chongqing 400712, China; National Citrus Engineering Research Center, Beibei, Chongqing 400712, China; National Citrus Improvement Center, Southwest University, Chongqing 400712, China; Citrus Research Institute, Southwest University/Chinese Academy of Agricultural Sciences, Beibei, Chongqing 400712, China; Citrus Research Institute, Southwest University/Chinese Academy of Agricultural Sciences, Beibei, Chongqing 400712, China; Citrus Research Institute, Southwest University/Chinese Academy of Agricultural Sciences, Beibei, Chongqing 400712, China; Citrus Research Institute, Southwest University/Chinese Academy of Agricultural Sciences, Beibei, Chongqing 400712, China; Citrus Research Institute, Southwest University/Chinese Academy of Agricultural Sciences, Beibei, Chongqing 400712, China; Citrus Research Institute, Southwest University/Chinese Academy of Agricultural Sciences, Beibei, Chongqing 400712, China; Citrus Research Institute, Southwest University/Chinese Academy of Agricultural Sciences, Beibei, Chongqing 400712, China; Citrus Research Institute, Southwest University/Chinese Academy of Agricultural Sciences, Beibei, Chongqing 400712, China; Citrus Research Institute, Southwest University/Chinese Academy of Agricultural Sciences, Beibei, Chongqing 400712, China; Citrus Research Institute, Southwest University/Chinese Academy of Agricultural Sciences, Beibei, Chongqing 400712, China; Citrus Research Institute, Southwest University/Chinese Academy of Agricultural Sciences, Beibei, Chongqing 400712, China; National Citrus Engineering Research Center, Beibei, Chongqing 400712, China; National Citrus Improvement Center, Southwest University, Chongqing 400712, China; Citrus Research Institute, Southwest University/Chinese Academy of Agricultural Sciences, Beibei, Chongqing 400712, China; National Citrus Engineering Research Center, Beibei, Chongqing 400712, China; National Citrus Improvement Center, Southwest University, Chongqing 400712, China; Citrus Research Institute, Southwest University/Chinese Academy of Agricultural Sciences, Beibei, Chongqing 400712, China; National Citrus Engineering Research Center, Beibei, Chongqing 400712, China; National Citrus Improvement Center, Southwest University, Chongqing 400712, China; Citrus Research Institute, Southwest University/Chinese Academy of Agricultural Sciences, Beibei, Chongqing 400712, China; National Citrus Engineering Research Center, Beibei, Chongqing 400712, China; National Citrus Improvement Center, Southwest University, Chongqing 400712, China; Citrus Research Institute, Southwest University/Chinese Academy of Agricultural Sciences, Beibei, Chongqing 400712, China; National Citrus Engineering Research Center, Beibei, Chongqing 400712, China; National Citrus Improvement Center, Southwest University, Chongqing 400712, China

## Abstract

As the bacterial etiologic agent causing citrus bacterial canker (CBC), *Xanthomonas citri* subsp. *citri* (*Xcc*) seriously impacts citrus plantation and fruit production globally. In an earlier study, we demonstrated that CsBZIP40 can positively impact CBC resistance in the sweet orange (*Citrus sinensis*). However, the mechanistic basis for the protective benefits conferred by CsBZIP40 is yet to be delineated. Here, we show that CsBZIP40 positively regulates CBC resistance and reactive oxygen species (ROS) homeostasis in transgenic sweet orange overexpressing CsBZIP40. CsBZIP40 directly binds to the TGA-box of the CsWRKY43 promoter to repress its transcriptional activity. CsWRKY43 overexpression induces CBC susceptibility in transgenic sweet oranges. In contrast, its inhibition produces strong resistance to CBC. CsWRKY43 directly binds to the W-boxes of the CsPrx53 and CsSOD13 promoters to positively regulate the activities of these antioxidant enzymes, resulting in the negative regulation of ROS homeostasis and CBC resistance in sweet orange plants. CsPrx53/CsSOD13 knockdown enhances ROS accumulation and CBC resistance. Overall, our results outline a regulatory pathway through which CsBZIP40 transcriptionally represses CsWRKY43-CsPrx53/CsSOD13 cascade-mediated ROS scavenging in a manner conducive to CBC resistance. These mechanisms underscore the potential importance of CsBZIP40, CsWRKY43, CsPrx53, and CsSOD13, providing promising strategies for the prevention of CBC.

## Introduction

Bacterial infections can cause serious damage to plants. Over the course of evolution, plants have developed several defense mechanisms to protect themselves against the deleterious effects of bacterial infections. Evidence shows that plant transcription factors (TFs) attenuate biotic stress through the activation of specific DNA sequences [1]. One example of such TFs is Basic Leucine Zipper (BZIP) TF, which have a conserved basic region and a leucine zipper that binds to DNA, and play a role in defense responses against abiotic and biotic stresses [2]. So far, BZIP TFs have been identified in several crops including rice, *Arabidopsis*, soybean, and sweet orange [2–5]. A large number of studies have also demonstrated the key roles of BZIP TFs in responses against plant pathogens and disease defense. For example, group D *Arabidopsis* BZIPs have been implicated in processes contributing to pathogen defense [3]. Similarly, it is known that GmbZIP15 promotes resistance against the bacterial pathogens *Sclerotinia sclerotiorum* and *Phytophthora sojae* [6], while GmbZIP19 regulates a plethora of biotic and abiotic stress responses in soybean [7]. Further, studies have shown that CabZIP2 can positively enhance disease resistance to bacterial pathogenic infections in pepper plants [8].

One key component of the response to pathogenic invasion in plants is the production of reactive oxygen species (ROS), which are important signaling molecules [9, 10]. In the absence of infection, ROS levels are maintained at low levels to avoid oxidative damage. However, following bacterial infections, the rapid accumulation of ROS can activate immune pathways, induce a hypersensitivity response (HR), or kill the bacteria [11–13]. BZIP TFs have been found to activate various phytohormone signaling pathways, ROS pathways, and multigenic regulatory networks in response to bacterial infection [14, 15] For instance, in *Arabidopsis thaliana*, AtBZIP10 is known to regulate pathogenesis-related (PR) gene expression, ROS-mediated cell death, and basal defense responses [16].

Citrus bacterial canker (CBC) is a grave plant disease caused by infection with *Xanthomonas citri* subsp. *citri* (*Xcc*) and represents a major threat to global citrus production [12, 17]. Molecular breeding efforts aimed at increasing disease resistance in plants currently represent a promising approach for controlling the spread of CBC. CsBZIP40, a *Citrus sinensis* (sweet orange) BZIP TF belonging to group D, has been demonstrated to be upregulated in CBC-resistant crops and unaltered in the CBC-susceptible variety [2]. In sweet orange, the overexpression of CsBZIP40 increases the resistance against CBC, while its knockdown renders transgenic plants more susceptible to the disease [2]. At present, how CsBZIP40 regulates CBC resistance remains to be fully demonstrated. A previous study showed that CsBZIP40 positively regulates CBC resistance by interacting with CsNPR1 and activating the expression of *PR* [2]. However, the role of CsBZIP40 in additional pathways warrants further investigation.

In this study, a series of transcriptomic analyses, molecular interaction assays, reverse genetics-based functional validation studies, functional recovery tests, and biochemical assays were performed to explore the mechanisms of CsBZIP40 in CBC resistance. The findings ultimately revealed a regulatory pathway through which CsBZIP40 confers CBC resistance by repressing CsWRKY43-CsPrx53/CsSOD13 cascade-mediated ROS scavenging. Together, the findings offer novel insights regarding the mechanistic effects of CsBZIP40, extending the potential utility of CsBZIP40, CsWRKY43, CsPrx53, and CsSOD13 as targets for breeding CBC-resistant varieties of citrus plants.

## Results

### CsBZIP40 positively regulates CBC resistance and promotes the altered expression of biotic stress-related genes and ROS homeostasis

**Figure 1:**
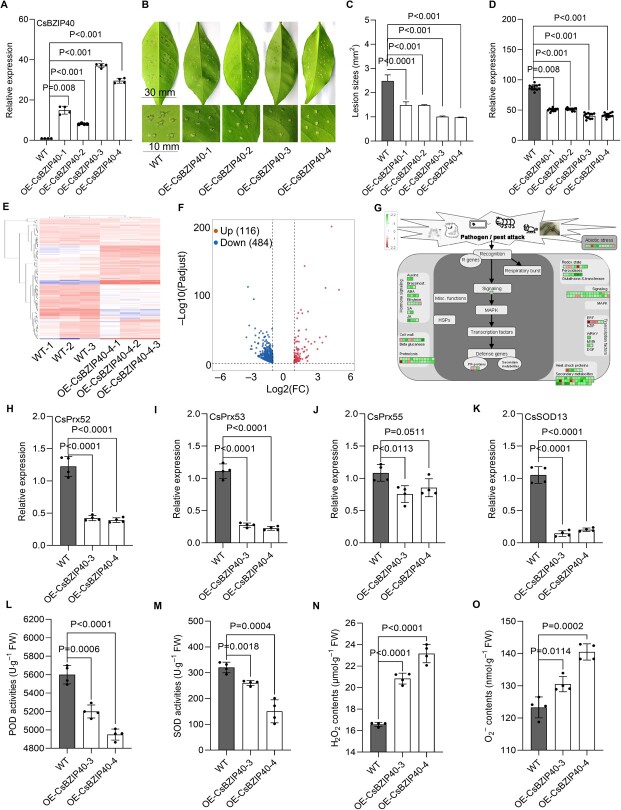
Assessment of CBC resistance, transcriptomic profiles, antioxidant enzymic activities, and ROS levels in CsBZIP40-overexpressing Wanjincheng plants. **A** Relative expression of CsBZIP40, with CsActin serving as the normalization control. **B***Xcc*-induced disease symptoms assessed in WT and CsBZIP40-overexpressing plants at 10 dpi. Scale bar = 30 mm (leaves) and 10 mm (leaf disks). **C** CBC lesion sizes quantified in WT and CsBZIP40-overexpressing leaves at 10 dpi (means ± SDs; *n* = 240). **D** CBC disease index values computed between WT and CsBZIP40-overexpressing plants at 10 dpi (means ± SDs; *n* = 8). **E** Correlations among samples (leaves) used for the transcriptomic assays, with three replicates for WT and CsBZIP40-overexpressing plants, respectively. **F** Volcano plots showing DEGs; 116 upregulated and 484 downregulated DEGs were visualized. **G** MapMan DEG clustering analyses used to analyse the biotic stress pathway based on the built-in *Citrus sinensis* reference database. Relative expression of CsPrx52 **(H)**, CsPrx53 **(I)**, CsPrx55 **(J)**, and CsSOD13 **(K)** in transgenic plants overexpressing CsBZIP40, with CsActin serving as the normalization control. POD **(L)** and SOD **(M)** activity levels analysed in WT and transgenic plants. H_2_O_2_**(N)** and O–2 **(O)** levels analysed in WT and transgenic plants. In (**A**) and (**H**)–(**O**), the data are provided as the means ± SDs (*n* = 4) and were compared using two-tailed *t*-tests. WT: wild type Wanjincheng; OE-CsBZIP40–1, 2, 3, and 4: transgenic Wanjincheng overexpressing CsBZIP40.

Transgenic citrus plants in which CsBZIP40 was overexpressed or knocked down were previously generated and used to explore the role of this gene in the context of *Xcc* infection. CBC resistance analyses conducted using 1-year-old transgenic plants ultimately revealed that CsBZIP40 overexpression was associated with significantly enhanced CBC resistance, whereas the silencing of this gene had the opposite effect, contributing to CBC susceptibility [2]. To further explore the stability, expression, and CBC resistance-related effects of CsBZIP40 in transgenic plants, we herein re-identified the presence and expression of CsBZIP40 and analysed the CBC resistance of 3-year-old transgenic plants. PCR confirmed that four transgenic plants (OE-CsBZIP40–1, 2, 3, and 4) produced a 608 bp fragment, which was absent in WT plants. Meanwhile, all the plants produced a 1068 bp fragment amplified from the genomic DNA (Fig. S2A, see online supplementary material). GUS staining revealed visible blue colorations on the leaf disc edges (Fig. S2B, see online supplementary material). As expected, qRT-PCR confirmed that the four transgenic plants expressed significantly higher levels of CsBZIP40 (15.0-fold, 8.2-fold, 37-fold, and 30.0-fold over WT, respectively) (Fig. 1A). The pinprick inoculation approach revealed that CBC symptoms – evaluated based on the CBC pustules – were less severe in these CsBZIP40-overexpressing transgenic plants than in WT controls (Fig. 1B). The lesions on OE-CsBZIP40–4 leaves were the smallest, only 39.3% of the size of WT lesions. Meanwhile, the leaves of OE-CsBZIP40–3 plants had slightly larger lesions (40.7% of WT) (Fig. 1C). In line with the lesion sizes, disease severity in transgenic plants – quantified based on the DI – was 41.1% (OE-CsBZIP40–2) and 52.6% (OE-CsBZIP40–3) lower than that in WT controls (Fig. 1D). These above-mentioned data proved that the overexpression of CsBZIP40 can enhance resistance to CBC in sweet orange plants. CsBZIP40 was thus identified as a persistent regulator of CBC resistance in *C. sinensis*.

Transcriptomic analyses were additionally used to explore the role of CsBZIP40 in *C. sinensis*. The WT wanjincheng plant and mixture of transgenic plants OE-CsBZIP40–3 and 4 were selected for the transcriptomic analyses (three replicates per sample). Correlation analyses revealed that transcriptional levels were clearly distinct between the WT and CsBZIP40-overexpressing plants (Fig. 1E). Overall, 116 upregulated DEGs and 484 downregulated DEGs were identified after comparing WT and CsBZIP40-overexpressing plants (Fig. 1F; Table S6, see online supplementary material). MapMan analyses of genes associated with the pathogen/pest attack pathway led to the identification of 176 DEGs associated with redox status, transcriptional regulation, hormone signaling, etc. (Fig. 1G). Among the DEGs, POD and SOD enzymes encoding genes *CsPrx52*, *CsPrx53*, *CsPrx55*, and *CsSOD13* were obtained, which were downregulated by CsBZIP40 overexpression, as detected by RNA-seq assay (Table S6, see online supplementary material). We further validated the transcriptomic data with qRT-PCR, and as expected, the results showed that transcriptions of genes *CsPrx52*, *CsPrx53*, *CsPrx55*, and *CsSOD13* were significantly downregulated in Wanjincheng overexpressing CsBZIP40 (Fig. 1H–K). Of the four genes, transcriptions of *CsPrx53* and *CsSOD13* were inhibited more strongly (Fig. 1I and K). The above findings suggest that CsBZIP40 can transcriptionally regulate genes encoding antioxidant enzymes such as SOD and POD, further affecting antioxidant enzyme activity and ROS homeostasis.

POD and SOD are key members of the enzymatic antioxidant system and are involved in ROS scavenging [9, 18]. Hence, we hypothesized that CsBZIP40 overexpression may increase ROS homeostasis within citrus plant cells in part by repressing antioxidant enzymes. To test this possibility, POD and SOD activities were evaluated in plants overexpressing CsBZIP40. Subsequently, we found that the activities of these enzymes were significantly lower in CsBZIP40-overexpressing plants than in WT plants (Fig. 1L–M). As expected, ROS, such as H_2_O_2_ and O–2, showed significantly higher levels in CsBZIP40-overexpressing as a consequence of impaired ROS scavenging (Fig. 1N–O). These findings suggest that CsBZIP40 can increase ROS levels by repressing the genes coding antioxidant enzymes. Overall, we concluded that CsBZIP40 positively regulates CBC resistance and promotes the altered expression of enzymatic antioxidant system and ROS homeostasis.

### CsBZIP40 directly binds to the *CsWRKY43* promoter to repress its transcription

**Figure 2:**
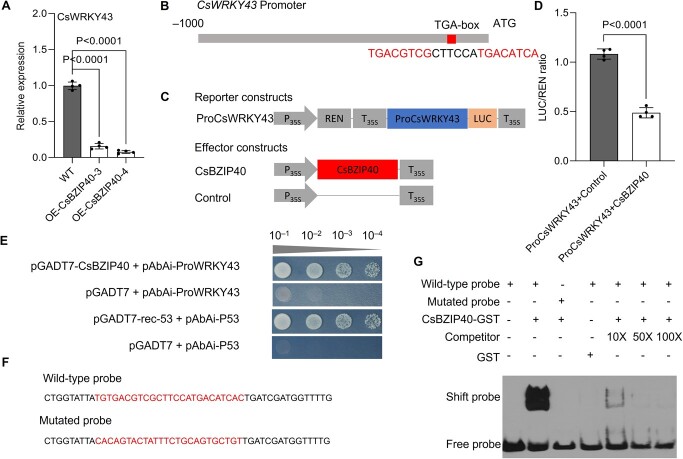
Analyses of the interaction between CsBZIP40 and the *CsWRKY43* promoter. **A** Relative expression of CsWRKY43 in transgenic plants overexpressing CsBZIP40 assessed using qRT-PCR, with CsActin serving as the normalization control. WT: wild type Wanjincheng; OE-CsBZIP40–3 and 4: transgenic Wanjincheng overexpressing CsBZIP40. Leaves were picked for all assays. **B** Putative binding site for CsBZIP40 in the *CsWRKY43* promoter predicted using JASPAR V2020. **C** Reporter and effector vectors utilized for dual-luciferase reporter assays. REN, Renilla luciferase; LUC, firefly luciferase; P_35S_, CaMV35S promoter; T_35S_, CaMV35S terminator. **D** Promoter activity analysed in tobacco leaves following the transient transfection of the indicated constructs, with data shown as the ratio of LUC to REN signal. In (**A**) and (**D**), the data are provided as the means ± SDs (*n* = 4) and were compared using two-tailed *t*-tests. **E** Y1H assays performed for assessing the interactions between CsBZIP40 and the *CsWRKY43* promoter using a gradient of yeast cultures (10^−1^ to 10^−4^). In (**C**)–(**E**), ProCsWRKY43: 1000-bp promoter of *CsWRKY43*. **F** WT and MT probes utilized in EMSA analyses. **G** EMSA results for the analyses of specific CsBZIP40 binding to the *CsWRKY43* promoter. CsBZIP40-GST was incubated with biotinylated probe constructs. +, presence; −, absence.

Transcriptomic analysis showed that *CsWRKY43* was the most highly downregulated DEG in plants overexpressing CsBZIP40 (Table S6, see online supplementary material), which was confirmed using qRT-PCR (Fig. 2A). We therefore sought to examine whether CsBZIP40 directly targets the *CsWRKY43* gene. Analyses of the *CsWRKY43* promoter revealed one candidate binding site with a relative score of 87.6% (Fig. 2B; Fig. S3A–C, see online supplementary material). To test the ability of CsBZIP40 to regulate *CsWRKY43* transcription, a Dual LUC assay was performed. In this assay, the identified promoter region was ligated into the pGreenII0800 vector upstream of the luciferase gene to produce a reporter construct (ProCsWRKY43). Meanwhile, in the effector construct, CsBZIP40 was under the CaMV35S promoter (Fig. 2C). These constructs were transiently expressed in tobacco leaves. The firefly luciferase signal induced in leaves transduced with both constructs was lower than that induced in leaves transfected with either construct alone (Fig. 2D). This suggests that CsBZIP40 could enhance *CsWRKY43* promoter activity. To validate this interaction, a Y1H assay was also performed. CsBZIP40 was cloned, and a prey construct was generated to confirm the ability of CsBZIP40 to interact with the *CsWRKY43* promotor. Self-activation tests revealed that the promoter of *CsWRKY43* had self-activating activity and needed to be inhibited with 100 ng·mL^−1^ of AbA (Fig. S4, see online supplementary material). The only yeasts that could grow in this Y1H assay system were those containing the pGADT7-CsBZIP40 and ProCsWRKY43 plasmids and the positive control yeast cells, confirming the ability of CsBZIP40 to directly bind to the *CsWRKY43* promoter region (Fig. 2E). An EMSA approach was also adopted to test this interaction *in vitro* using WT and MT probes designed based on the predicted binding site (Fig. 2B and F). When the purified CsBZIP40 protein was incubated with the WT probe, the migration of the resultant protein-DNA complex was retarded. The size of the band decreased when the amount of unlabeled WT competitor probe increased, and the band disappeared completely when the concentration of the competitor became 100-fold higher than that of the labeled WT probe. In contrast, the MT probe harboring mutations in its core binding site motif failed to interact with *CsWRKY43* promoter. Hence, no effect on the speed of migration was observed. These findings confirmed the ability of the core binding site in the WT probe to interact with *CsWRKY43* promoter (Fig. 2G). In conclusion, the results demonstrate that CsBZIP40 is an upstream TF that directly binds to the *CsWRKY43* promoter to repress its transcriptional activity.

### CsWRKY43 is induced by *Xcc*, localizes to the nucleus and possesses transcriptional activities

**Figure 3:**
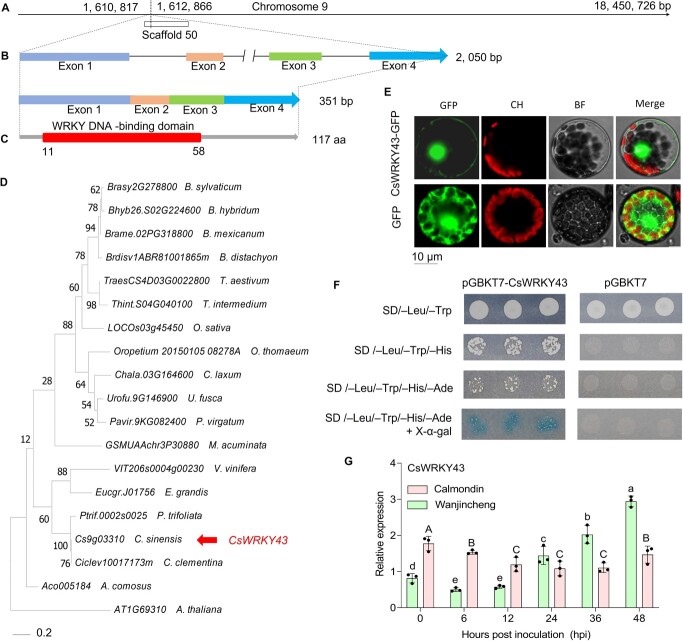
Bioinformatics analyses and characterization of CsWRKY43 expression. **A** The chromosomal locus encoding CsWRKY43 as identified using CPDB. **B** The *CsWRKY43* exon-intron structure as determined using GSDS V2.0. bp: base pair. **C** CsWRKY43 functional domains identified using HMMER. aa: amino acid. **D** Maximum-likelihood phylogenetic analyses of CsWRKY43 and orthologous proteins performed using MEGA X (bootstrap: 500, Poisson model). Branches are drawn to scale, and the branch length is indicative of the number of substitutions at each site. Protein IDs and source species are listed to the right of the evolutionary tree. **E** Subcellular CsWRKY43 localization within *Arabidopsis thaliana* protoplasts. GFP, chlorophyll autofluorescence (CH), bright field (BF), and merged images (MERGE) are shown. Scale bar = 10 μm. **F** Transcriptional activation of CsWRKY43 in yeast. **G** CsWRKY43 expression in Wanjincheng and Calamondin leaves following *Xcc* infection assessed using qRT-PCR, with CsActin as the normalization control. Data were compared using ANOVA and Duncan’s multiple range test.

Next, the full-length ORF of Wanjincheng *CsWRKY43* was amplified and sequenced. Sequence similarity and conserved domain analyses were performed, revealing that this gene belonged to the same cluster as the *Cs9g03310* gene in CPBD (Table S2, see online supplementary material). *CsWRKY43* was located on Chromosome 9 of *C. sinensis* (Fig. 3A). *CsWRKY43* contained four exons encoding a 351 amino acid WRKY family TF and harbored a classic WRKY DNA-binding domain (Fig. 3B–C). Phylogenetic comparisons of this protein and orthologs from other species revealed a close relationship between CsWRKY43, a *Populus trichocarpa* WRKY TF, and a *Citrus clementina* WRKY TF (Fig. 3D; Table S7, see online supplementary material). The transient expression of GFP-tagged CsWRKY43 mainly produced a GFP signal in the nucleus. In contrast, the control signal was detectable in both the nucleus and cytosol. These findings further confirmed the identity of CsWRKY43 as a nuclear protein (Fig. 3E). A small amount of fluorescence was seen on the cell membrane of GFP-tagged CsWRKY43-expressing cells, indicating that CsWRKY43 could localize to the cell membrane or interact with proteins present on the cell membrane (Fig. 3E). To assess the ability of CsWRKY43 to activate transcription, the full-length ORF of *CsWRKY43* was introduced into the pGBKT7 vector downstream of GAL4BD, and the resultant construct was used to transform Y2HGold yeast cells. These yeast cells grew normally on synthetic dropout medium lacking tryptophan (SDO), but only GAL4BD-CsWRKY43 vector-transformed cells were able to survive and turn blue on selective medium containing X-α-gal and AbA (SDO/X/AbA) (Fig. 3F). Hence, the GAL4BD-CsWRKY43 fusion protein could activate the MEL1 and AUR1 reporter genes, indicating that CsWRKY43 possesses the potential to activate transcription. To examine the association between CsWRKY43 and CBC resistance, *Xcc-*induced changes in CsWRKY43 expression were analysed. CsWRKY43 was found to be downregulated in the CBC-resistant Calamondin variety in response to *Xcc* infection. However, its expression levels were elevated in the CBC-susceptible Wanjincheng variety after *Xcc* infection (Fig. 3G). Together, these bioinformatics and cellular assays successfully validated that CsWRKY43 is a promising target for further studies on the regulation of CBC resistance.

### Overexpression of CsWRKY43 confers CBC susceptibility, while CsWRKY43 silencing confers CBC resistance

**Figure 4:**
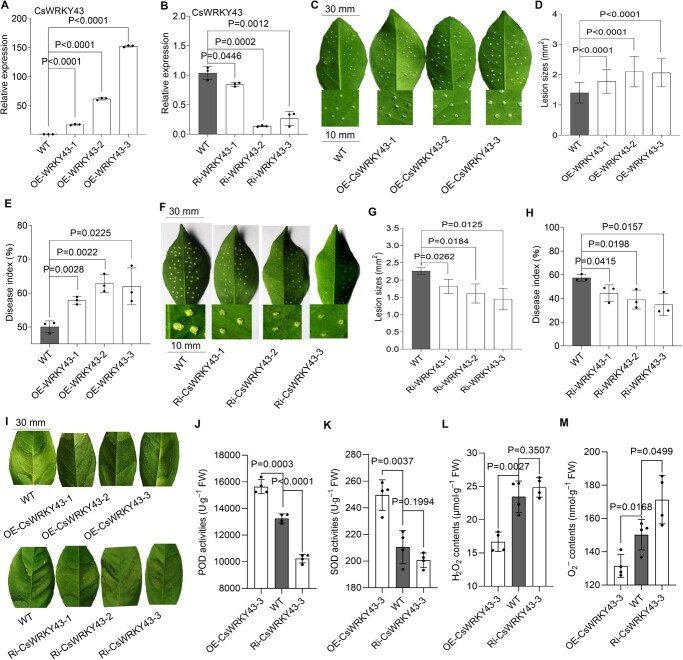
Assessment of CBC resistance and biochemical assays in CsWRKY43 transgenic plants. **A** CsWRKY43 expression in CsWRKY43-overexpressing transgenic plants. **B** CsWRKY43 expression in CsWRKY43-RNAi transgenic plants. In (**A**) and (**B**), CsActin was considered as the normalization control. **C***Xcc*-induced disease symptoms assessed in WT and CsWRKY43-overexpressing plants at 10 dpi. Scale bar = 30 mm for leaves and 10 mm for leaf disks. **D** CBC lesion sizes in WT and CsWRYK43-overexpressing plants analysed at 10 dpi. **E** CBC disease index values in WT and CsWRYK43-overexpressing plants assessed at 10 dpi. **F***Xcc*-induced disease symptoms assessed in WT and CsWRKY43-RNAi plants at 10 dpi. Scale bar = 30 mm for leaves and 10 mm for leaf disks. **G** CBC lesion sizes in WT and CsWRYK43-RNAi plants analysed at 10 dpi. **H** CBC disease index values in WT and CsWRYK43-knockdown plants assessed at 10 dpi. **I** Symptoms of *Xcc*-induced disease in WT and CsWRYK43-overexpressing and knockdown plants assessed at 10 dpi via the infiltration method. Scale bar = 30 mm. POD (**J**) and SOD (**K**) activity levels analysed in WT and transgenic plants. H_2_O_2_ (**L**) and O–2 (**M**) levels analysed in WT and transgenic plants. Data in (**A**) and (**B**), (**E**), (**H**), and (**J**)–(**M**) are provided as the means ± SDs (*n* = 4) and were compared using two-tailed *t*-tests. Data in (**D**) and (**G**) are provided as the means ± SDs (*n* = 120) and were compared using two-tailed t-tests. WT: wild type Wanjincheng; OE-CsWRKY43–1, 2, and 3: transgenic Wanjincheng overexpressing CsWRKY43. Ri-CsWRKY43–1, 2, and 3: transgenic Wanjincheng repressing CsWRKY43.

Several gain- and loss-of-function experiments were conducted to confirm the role of CsWRKY43 as a mediator of CBC resistance in sweet orange plants. Six transgenic plants, including three CsWRKY43-overexpressing plants and three CsWRKY43-RNAi plants, were generated (Fig. S5A, see online supplementary material). Using PCR, a 500 bp fragment was detected in CsWRKY43-overexpressing plants and CsWRKY43-RNAi citrus plants (Fig. S5B, see online supplementary material). Except for Ri-CsWRKY43–3, which had a low growth rate, the other transgenic plants had a growth rate similar to wild-type plants (Fig. S5C, see online supplementary material). qRT-PCR confirmed that the CsWRKY43-overexpressing plants expressed significantly elevated levels of CsWRKY43 (17.7-fold, 62.1-fold, and 152.8-fold over WT, respectively) (Fig. 4A), while CsWRKY43-RNAi plants expressed significantly lower CsWRKY43 levels (84.6%, 13.8%, and 26.9% of WT, respectively) (Fig. 4B).

The pinprick inoculation approach revealed that CBC symptoms (i.e. CBC pustules) were much more severe in CsWRKY43-overexpressing transgenic plants than in WT controls (Fig. 4C). The lesions on CsWRKY43–2 leaves were the largest, being ~1.50 times the size of WT lesions. Meanwhile, slightly smaller lesions were detected on the leaves of OE-CsWRKY43–3 (1.47-fold of WT) and OE-CsWRKY43–1 plants (1.27-fold of WT) (Fig. 4D). Disease severity was quantified based on the DI, and it was found to be 15.5% (OE-CsWRKY43–1) to 25.5% (OE-CsWRKY43–2) higher in transgenic plants than in WT controls (Fig. 4E). CBC pustules indicated that the CBC symptoms in CsWRKY43-RNAi plants were less severe than those in WT controls, with the greatest resistance being observed in Ri-CsWRKY43–3, followed by Ri-CsWRKY43–2 and Ri-CsWRKY43–1 (Fig. 4F). The lesions on CsWRKY43–3 leaves were the smallest at ~64.1% of the size of WT lesions, with slightly larger lesions being detected on the leaves of Ri-CsWRKY43–2 (71.2% of WT) and Ri-CsWRKY43–1 plants (80.1% of WT) (Fig. 4G). Disease severity quantified based on the DI was 22.6% (Ri-CsWRKY43–1) to 39.4% (Ri-CsWRKY43–3) lower in transgenic plants relative to WT controls (Fig. 4H). The infiltration method revealed clearly visible pustules at sites of infection at 10 dpi in transgenic plants overexpressing CsWRKY43, while a marked reduction in canker symptoms was seen in CsWRKY43-repressed plants (Fig. 4I). Overall, CsWRKY43 overexpression strongly enhanced resistance to CBC in transgenic sweet orange plants, while CsWRKY43 silencing had the opposite effect.

We showed that CsBZIP40 negatively regulates antioxidant enzymes, leading to an increase in ROS content. As *CsWRKY43* is a target gene negatively regulated by CsBZIP40, it is worth exploring whether CsWRKY43 itself affects antioxidant enzymes and ROS contents. We found that in CsWRKY43-overexpressing plants, the POD and SOD activities were significantly increased, while in CsWRKY43-RNAi plants, the two enzymes showed significantly reduced activity (Fig. 4J and K). As expected, H_2_O_2_ and O–2 levels were significantly elevated as a consequence of impaired ROS scavenging (Fig. 4L and M). These findings suggest that CsWRKY43 acts as an activator of antioxidant enzymes, thus contributing to ROS scavenging.

### CsWRKY43 directly binds to the *CsPrx53* and *CsSOD13* promoters to induce transcriptional activity

**Figure 5:**
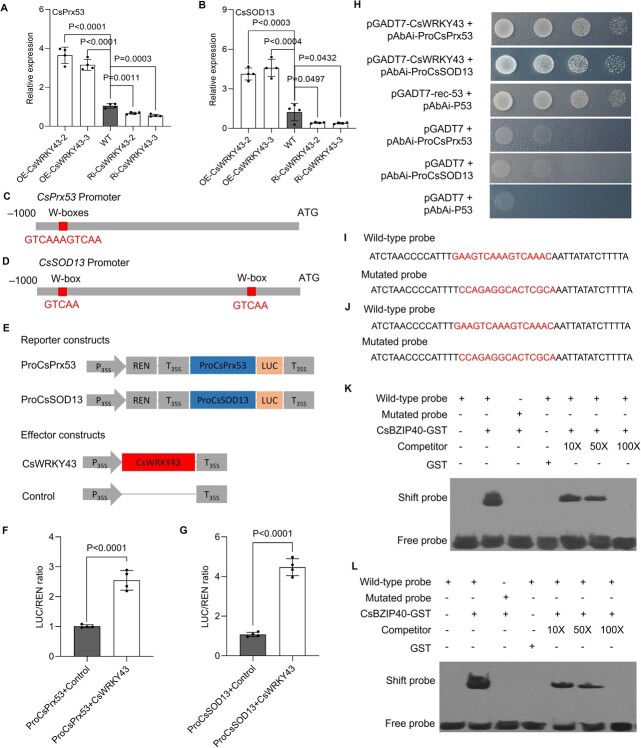
Analyses of the interaction between CsWRKY43 and the *CsPrx53*/*CsSOD13* promoters. **A** Relative expression of CsPrx53 in transgenic plants with CsWRKY43 overexpression and silencing; CsActin served as the normalization control. **B** Relative expression of CsSOD13 in transgenic plants with CsWRKY43 overexpression and silencing assessed using qRT-PCR; CsActin served as the normalization control. In (**A**) and (**B**), WT: wild type Wanjincheng; OE-CsWRKY43–2 and OE-CsWRKY43–3: transgenic Wanjincheng overexpressing CsWRKY43; Ri-CsWRKY43–2 and Ri-CsWRKY43–3: transgenic Wanjincheng suppressing CsWRKY43. Leaves were picked for all assays. **C** Putative binding site for CsWRKY43 on the *CsPrx53* promoter predicted using JASPAR V2020. **D** Putative binding site of CsWRKY43 on the *CsSOD13* promoter predicted using JASPAR V2020. **E** The reporter and effector vectors utilized for dual-luciferase reporter assays. REN, Renilla luciferase; LUC, firefly luciferase; P_35S_, CaMV35S promoter; and T_35S_, CaMV35S terminator. **F** and **G** Promoter activity analysed in tobacco leaves following the transient transfection of the indicated constructs, with data shown as the ratio of LUC to REN signal. In (**A**), (**B**), (**F**) and (**G**), the data are provided as the means ± SDs (*n* = 4) and were compared using two-tailed *t*-tests. **H** Y1H assays performed for assessing the interactions between CsWRKY43 and the *CsPrx53*/*CsSOD13* promoters using a gradient of yeast cultures (10^−1^ to 10^−4^). In (**E**)–(**H**), ProCsPrx53: 1000-bp promoter of *CsPrx53*; ProCsSOD13: 1000-bp promoter of *CsSOD13*. **I** WT and MT probes utilized in the EMSA of the CsWRKY43 and *CsPrx53* promoter, with red indicating the mutated binding sites. **J** WT and MT probes utilized in the EMSA of the CsWRKY43 and *CsSOD13* promoter. **K** EMSA results for the analyses of specific CsWRKY43 binding to the *CsPrx53* promoter. **L** EMSA results for the analyses of specific CsWRKY43 binding to the *CsSOD13* promoter. In (**K**) and (**L**), CsWRKY43-GST was incubated with biotinylated probe constructs. +, presence; −, absence.

Given that CsWRKY43 positively regulates POD and SOD activity (Fig. 4J and K), and that CsBZIP40 exerts inhibitory effects on CsWRKY43 (Fig. 2A) as well as POD and SOD activity (Fig. 1L and M), we speculated that CsWRKY43 may positively regulate CsPrxs and CsSOD13 downstream of CsBZIP40. Significant increases in CsPrx53 and CsSOD13 expression were observed in CsWRYK43-overexpressing plants, while their expression was reduced in CsWRYK43-RNAi plants (Fig. 5A and B). Hence, we cloned and analysed the promoters of *CsPrxs* and *CsSOD13* and studied their interactions with CsWRKY43. Two adjacent WRKY binding sites (W-boxes) were detected in the CsPrx53 promoter (Fig. 5C; Fig. S6A, see online supplementary material), showing high relative scores (>90.0%) with the binding sites in CsWRKY43 homologs, while no CsBZIP40 binding elements in the promoters of CsPrxs and CsSOD13 (Fig. S6B and C, see online supplementary material). This indicated a high probability of binding between CsWRKY43 and the CsPrx53 promoter. Meanwhile, two separate W-boxes were detected in the CsSOD13 promoter (Fig. 5D; Fig. S7A, see online supplementary material), showing high relative scores (>85.0%) with the binding sites in CsWRKY43 homologs, while also no CsBZIP40 binding elements in the promoters of CsPrxs and CsSOD13 (Fig. S7B and C, see online supplementary material). To test the ability of CsWRYK43 to regulate *CsPrx53* and *CsSOD13* transcription, a Dual LUC assay was performed. The identified promoter region was inserted into the pGreenII0800 vector upstream of the luciferase gene to produce reporter constructs (ProCsPrx53 and ProCsSOD13). Additionally, the effector construct contained CsWRYK43 under the control of the CaMV35S promoter (Fig. 5E). When these constructs were transiently expressed in tobacco leaves, a stronger firefly luciferase signal was induced in leaves transduced with both constructs than in leaves transfected with either construct alone (Fig. 5F and G). This suggests that CsWRYK43 can activate *CsPrx53* and *CsSOD13* promoters. CsWRYK43 was then cloned, and a prey construct was generated prior to a Y1H assay for confirming the ability of CsWRYK43 to interact with the *CsPrx53* and *CsSOD13* promoters. Self-activation tests revealed that both the promoters of *CsPrx53* and *CsSOD13* show self-activating effects and need to be inhibited with 100 ng·mL^−1^ of AbA (Fig. S8A and B, see online supplementary material). The only yeast cells that could grow in this assay system were those containing the pGADT7-CsWRYK43 and ProCsPrx53/ProCsSOD13 plasmids and the positive control yeast, confirming the ability of CsWRYK43 to directly bind to the *CsPrx53* and *CsSOD13* promoter regions (Fig. 5H). GST-tagged CsWRKY43 was expressed in a prokaryote and purified (Fig. 5I). EMSA probes were generated based on WT or MT versions of the binding sites in the *CsPrx53* and *CsSOD13* promoters (Fig. 5J and K). When purified CsWRKY43 was incubated with the WT probe of CsPrx53, the resultant protein-DNA complex showed slower migration on electrophoresis. The size of the band decreased when the amount of unlabeled WT competitor probe was increased, and the band seemingly disappeared when the competitor concentration was 100-fold higher than that of the labeled WT probe. In contrast, the MT probe harboring mutations in its core binding site motif failed to interact with CsWRKY43. Hence, no effects on migration were observed during electrophoresis (Fig. 5L). The EMSA for CsWRKY43 and *CsSOD13* promoter yielded similar results (Fig. 5M). These findings thus confirmed the ability of the core binding sites in the WT probe to interact with CsWRKY43. In conclusion, CsWRKY43 acts as an upstream TF and directly binds to the *CsPrx53* and *CsSOD13* promoters to enhance their transcriptional activities.

### CsPrx53 and CsSOD13 are enzymes engaged in ROS scavenging

Next, the full-length ORF of Wanjincheng *CsPrx53* was amplified and sequenced. Sequence similarity and conserved domain analyses were performed and revealed that it clustered with the *orange1.1 t02041* genein CPBD (Table S3, see online supplementary material). On one hand, *CsPrx53* was located on the unassembled chromosome of *C. sinensis*. *CsPrx53* contained four exons encoding a 349 amino acid peroxidase family protein, harboring a peroxidase domain and an N-terminal signal peptide (Fig. S9, see online supplementary material). On the other hand, *CsSOD13* was part of the same cluster as the *Cs8g15520* gene in CPBD (Table S4, see online supplementary material). *CsSOD13* was located on chromosome 8 of *C. sinensis*. It also contained four exons, which encoded a 252 amino acid SOD family harboring a peroxidase domain and an N-terminal signal peptide (Fig. S10, see online supplementary material). Expression of CsPrx53 and CsSOD13 in the Wanjincheng (CBC sensitive) and Calamondin (CBC resistant) varieties was evaluated following *Xcc* infection to assess the relationships with CBC. CsPrx53 was significantly downregulated in Wanjincheng during 0–48 hours, while overall was up-induced in Wanjincheng (Fig. S11A, see online supplementary material). Overall, CsSOD13 showed similar *Xcc*-induciable patterns with CsPrx53 (Fig. S11B, see online supplementary material). These expression patterns are highly consistent with the *Xcc*-inducible expression pattern of CsWRKY43, and overall reverse to CsBZIP40 reported previously [2].

**Figure 6:**
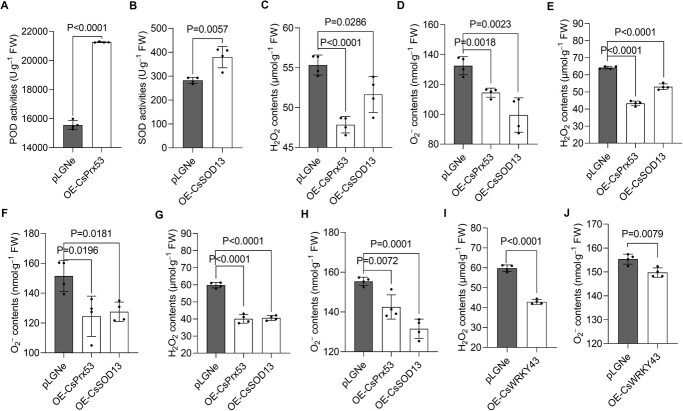
Transient overexpression assays of CsPrx53 and CsSOD13. **A** POD activities of plants after the transient overexpression of CsPrx53 in Wanjincheng plants. **B** SOD activities of plants after the transient overexpression of CsSOD13 in Wanjincheng plants. H_2_O_2_ and O–2 levels of plants after the transient overexpression of CsPrx53 (**C**) and CsSOD13 (**D**) in Wanjincheng plants. H_2_O_2_ (**E**) and O–2 (**F**) levels of plants after the transient overexpression of CsPrx53 and CsSOD13 in CsBZIP40-overexpressing Wanjincheng plants OE-CsBZIP40–4. H_2_O_2_ (**G**) and O–2 (**H**) levels of plants after the transient overexpression of CsPrx53 and CsSOD13 in CsWRKY43-silencing Wanjincheng plants Ri-CsWRKY43–3. H_2_O_2_ (**I**) and O–2 (**J**) levels of plants after the transient overexpression of CsWRKY43 in CsBZIP40-overexpressing Wanjincheng plants OE-CsBZIP40–4. In (**A**)–(**J**), the data are provided as the means ± SDs (*n* = 4) and were compared using two-tailed *t*-tests. In (**A**)–(**I**), OE-CsPrx53: CsPrx53 transient overexpression plants; OE-CsSOD13: CsSOD13 transient overexpression plants; pLGNe: plants transformed with the empty vector pLGNe taken as the controls.

To investigate the functions of CsPrx53 and CsSOD13 in resistance/susceptibility to CBC and ROS homeostasis regulation, we conducted experiments involving transient gene overexpression and VIGS. Compared to Wanjincheng plants transfected with an empty vector, plants with transient CsPrx53 overexpression exhibited increased POD activity (1.37-fold) (Fig. 6A). Meanwhile, CsSOD13 overexpression resulted in increased POD activity (1.34-fold) (Fig. 6B). Both CsPrx53 and CsSOD13 contributed to a reduction in H_2_O_2_ and O–2 levels. CsPrx53 was better at clearing H_2_O_2_, while CsSOD13 was more inclined to clear O–2, which indicates that CsPrx53 and CsSOD13 have different scavenging abilities for different ROS components (Fig. 6C and D). Our earlier experiments confirmed that CsBZIP40 overexpression inhibits the expression of CsPrx53 and CsSOD13 and increases ROS concentrations (Fig. 1I, K, N and O). To prove that CsPrx53 and CsSOD13 are regulators of the ROS balance in this context, we transiently overexpressed CsPrx53 and CsSOD13 in CsBZIP40-overexpressing plants. We found that both CsPrx53 and CsSOD13 overexpression attenuated H_2_O_2_ and O–2 levels in CsBZIP40-overexpressing plants (Fig. 6E and 6F). Similarly, the transient overexpression of CsPrx53 and CsSOD13 could inhibit the increase in ROS induced by CsWRKY43 silencing (Fig. 6G and H). Moreover, the transient overexpression of CsWRKY43 could also reverse the increase in ROS caused by CsBZIP40 overexpression (Fig. 6I and J). These results show that CsPrx53 and CsSOD13 are negatively regulated and positively regulated by CsBZIP40 and CsWRKY43, respectively, and this pathway plays a role in ROS homeostasis.

### Silencing of CsPrx53/CsSOD13 confers CBC resistance

**Figure 7:**
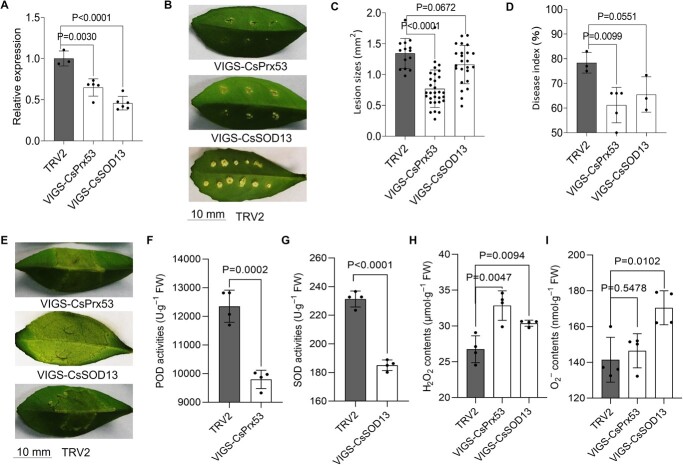
Assessment of CBC resistance, antioxidant enzymic activities and ROS levels in CsPrx53/CsSOD13-silencing Wanjincheng plants. **A** Relative expression of CsPrx53 and CsSOD13 determined using qRT-PCR and normalized based on CsActin levels. **B** Lesions in VIGS plants. Scale bar = 10 mm. **C** Lesion sizes in VIGS plants. **D** Disease index of VIGS plants. In (**B**)–(**D**), the VIGS plants were inoculated with *Xcc* at 10 dpi. **E** Symptoms in silenced plants. Scale bar = 10 mm. **F** POD activities in CsPrx53 VIGS plants. **G** SOD activities of CsSOD13 VIGS plants. H_2_O_2_ (**H**) and O–2 (**I**) levels in CsPrx53/CsSOD13 VIGS plants. In (**A**), (**D**), and (**F**)–(**I**), the data are provided as the means ± SDs (*n* = 4) and were compared using two-tailed *t*-tests. In (**C**), the data are provided as the means ± SDs (*n* = 30). In (**A**)–(**I**), VIGS-CsPrx53: CsPrx53 VIGS plants; VIGS-CsSOD13: CsSOD13 VIGS plants; TRV2: plants transformed with the empty vector TRV2, taken as controls.

CsPrx53 and CsSOD13 were next silenced in the Wanjincheng variety to further elucidate the roles of the two enzymes in CBC resistance. The plasmids for the VIGS of CsPrx53 and CsSOD13 were constructed using the TRV2 vector (Fig. S12, see online supplementary material). The success of VIGS in the plants was verified using PCR (Fig. S13, see online supplementary material), and the plants with VIGS exhibited relatively lower CsPrx53 and CsSOD13 expression than the controls (Fig. 7A). CBC resistance in the plants with VIGS was investigated after *Xcc* inoculation. Interestingly, these plants showed significantly less severe symptoms than the control (Fig. 7B). The lesion sizes were lower in both the CsPrx53- and CsSOD13-silenced plants (approximately 41.1% and 50.1% of the size of the control lesions, respectively) (Fig. 7C). The DI was 44.1% and 56.3% lower in the CsPrx53- and CsSOD13-silenced plants than in the control, respectively (Fig. 7D). The infiltration method revealed that at 10 dpi, there were clearly visible pustules at the sites of infection in control plants, while a marked reduction in canker symptoms was observed in CsPrx53/CsSOD13-repressed plants (Fig. 7E). These findings indicate that the silencing of CsPrx53 and CsSOD13 enhances resistance to CBC. POD and SOD activities were observed to be downregulated in plants with the VIGS of both CsPrx53 and CsSOD13 (Fig. 7F and G), and these plants also showed elevated H_2_O_2_ and O–2 levels (Fig. 7H and I). This indicates an association between increased CBC resistance in VIGS plants and the restoration of H_2_O_2_ homeostasis under regulation by CsPrx53 and CsSOD13.

## Discussion

### CsBZIP40 confers CBC resistance and induces ROS accumulation

ROS production is a consequence of aerobic respiration and often occurs after environmental alterations [19]. H_2_O_2_ is produced in response to a variety of stimuli and functions as a signaling molecule, influencing various processes such as plant growth, stress adaptation, development, and death. However, ROS can pass through membranes and can also damage various cellular components. Antioxidant mechanisms thus represent a critical component of plant adaptations to environmental stress. POD and SOD are key antioxidant enzymes and can maintain ROS homeostasis and protect cells from damage [9]. H_2_O_2_ is a highly abundant ROS intermediary and serves as an important signaling molecule [20], regulating immune activity through multiple mechanisms: (i) the H_2_O_2_ generated by respiratory burst oxidase homolog (RBOH) proteins can pass through aquaporins on the cell membrane [11, 21], thereby regulating immunity in plants via the induction of a hypersensitivity response; (ii) H_2_O_2_ signals interact with other signaling pathways, including those regulated by SA and abscisic acid (ABA), to facilitate the induction of systemic acquired resistance (SAR) [13, 22]; and (iii) H_2_O_2_ is capable of binding to cell membrane receptor kinases, activating Ca^2+^ channels in local or distant cells and inducing a Ca^2+^ influx, thereby initiating intracellular immunity [23–25]. We have also previously shown that in transgenic citrus plants, CsBZIP40 overexpression is associated with improved *Xcc* resistance. Meanwhile, the silencing of this gene has the opposite effect [26]. As such, CsBZIP40 seems to be a positive regulator of CBC resistance. Notably, following transcriptomic analyses, both POD and SOD were found to be downregulated in the plants overexpressing CsBZIP40, while the ROS levels in these plants were found to be enhanced (Fig. 1). These results highlighted the importance of the CsBZIP40-CsPrx53/CsSOD13 pathway as a potential regulator of ROS accumulation in plants.

### CsBZIP40 attenuates CsWRKY43-activated ROS scavenging

The exact mechanisms through which CsBZIP40 controls CBC resistance have so far been unclear. In general, TFs function by regulating target gene expression under stressful conditions [27]. Typically, multiple TFs control downstream genes in a regulatory cascade, as in the case of the PalWRKY77-PalNAC002 cascade that controls salt tolerance and ABA signaling in *Populus* [28]. Given that CsBZIP40 can promote *CsPrx53*/*CsSOD13* transcription in plants when overexpressed (Fig. 1), it can be concluded that CsBZIP40 and/or other transcriptional regulators must function upstream of CsPrx53 and CsSOD13. When we analysed the promoters of *CsPrx53* and *CsSOD13*, we failed to identify any complementary CsBZIP40 binding sites. However, clear binding sites for WKRY TFs could be detected. These results, together with the previous transcriptomic findings, revealed that the TF CsWRKY43 was the most significantly repressed gene in CsBZIP40-overexpressing plants (Fig. 1). Further, it was predicted to bind to the promoters of *CsPrx53* and *CsSOD13*. We therefore hypothesized that CsWRKY43 bridges the transcriptional regulation of CsBZIP40 to that of CsPrx53 and CsSOD13. This hypothesis was subsequently confirmed through Y1H, EMSA, and Dual-LUC (Figs 2 and 5) assays, validating the ability of CsBZIP40 to directly bind to and repress the *CsWRKY43* promoter. The analyses also demonstrated that CsWRKY43 in turn binds to and activates the promoters of *CsPrx53* and *CsSOD13*. Hence, based on the above analyses of *Xcc* growth and disease onset in CsWRKY43-overexpressing and -repressing plants, we conclude that CsWRKY43 can function as a negative regulator of CBC resistance in sweet oranges (Fig. 4). To critically demonstrate the above conclusions, we also examined the effect of CsBZIP40 silencing on downstream CsWRKY43, CsPrx53, and CsSOD13, which showed that CsBZIP40 silencing increased the expression levels of all three genes (Fig. S14A–C, see online supplementary material). Our reverse genetics and molecular interaction studies thus demonstrate that CsBZIP40 attenuates CsWRKY43-activated ROS scavenging to enhance ROS accumulation and CBC resistance in citrus plants.

### Proposed model of CsBZIP40-mediated CBC resistance

**Figure 8:**
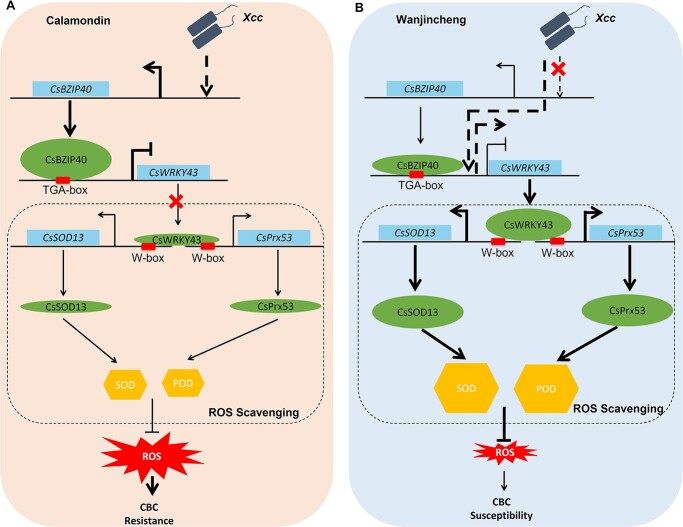
Mechanisms governing the levels of CBC resistance by ROS control in Calamondin and Wanjincheng plants. **A** Calamondin (citrus bacterial canker resistant variety). **B** Wanjincheng (citrus bacterial canker susceptible variety). Arrows denote activation, while flat lines represent inhibitory relationships. Direct and indirect regulation are respectively represented using solid and dashed lines. Ovals represent proteins. Crosses represent the blocking or weakening of regulatory pathways. Hexagons represent antioxidant enzymes. CBC: citrus bacterial canker; POD: peroxidases; ROS: reactive oxygen species; SOD: superoxide dismutase; *Xcc*: *Xanthomonas citri* subsp. *citri*. Thick arrows or thick flat heads indicate that activation or inhibition is intensified.

In CBC-resistant Calamondin plants, *Xcc* inoculation leads to CsBZIP40 upregulation. However, this alteration is not observed in the CBC-susceptible Wanjincheng variety [2]. Previous studies have demonstrated that CsBZIP40 can enhance CBC resistance by interacting with CsNPR1 and by upregulating the expression of the CsPR4 protein [2]. This study is an expansion of the regulatory network of CsBZIP40. In light of these results and the above functional assays, we propose a model through which this signaling pathway can govern the acquisition of CBC resistance in citrus plants. In Calamondin plants, infection with *Xcc* promotes CsBZIP40 upregulation, resulting in lower levels of CsWRKY43 expression and a consequent reduction in CsPrx53/CsSOD13-mediated ROS scavenging. This promotes ROS accumulation and CBC resistance. Similarly, in Wanjincheng plants, CsBZIP40 overexpression can repress CsWRKY43-CsPrx53/CsSOD13-mediated ROS scavenging and CBC susceptibility (Fig. 8A). In contrast, in susceptible Wanjincheng plants and plants in which CsBZIP40 has been knocked down, CsBZIP40 is not upregulated following *Xcc* inoculation. As a result, the CsBZIP40-CsWRKY43-CsPrx53/CsSOD13 pathway is not activated, ultimately resulting in susceptibility to CBC (Fig. 8B). It is worth mentioning that in Wanjincheng plants, CsWRKY43 – and not CsBZIP40 – is induced by *Xcc*, which further reduces the levels of ROS and CBC resistance in these plants. These findings also suggest that in addition to CsBZIP40 as an inhibitor of CsWRKY43, other TFs may also positively regulate the expression of CsWRKY43 in Wanjincheng (Fig. 8B).

While the overexpression of CsBZIP40 markedly enhanced the ability of Wanjincheng citrus plants to resist CBC, the resistance of CsBZIP40-overexpressing Wanjincheng plants was still inferior to that of Calamondin plants. This suggested that Calamondin citrus plants may engage other mechanisms to further augment their CBC resistance. Even so, these results highlight a novel approach for enhancing the CBC resistance of otherwise susceptible citrus varieties. CBC resistance can be increased to a higher degree by polygenic co-expression/inhibition. Hence, future research will be vital for expanding on these results by further analysing other genes that are regulated by CsBZIP40 and are involved in disease resistance mechanisms.

In summary, the present study offers new insight into the mechanisms through which CsBZIP40 and associated factors can regulate CBC resistance in sweet oranges. Strikingly, our results show that the CsBZIP40-CsWRKY43 cascade can confer resistance to CBC through the transcriptional reprogramming of CsPrx53/CsSOD13-mediated ROS homeostasis. The findings extend our current knowledge regarding the functions of BZIP family members and the TF cascades that govern phytopathogen resistance, underscoring the potential importance of CsBZIP40, CsWRKY43, CsPrx53, and CsSOD13 in future efforts aimed at breeding CBC-resistant citrus varieties.

## Materials and methods

### Plants and pathogens

The citrus trees utilized in this research were obtained from the National Citrus Germplasm Repository in Chongqing, China. These included the wild-type (WT) Calamondin (*Citrus madurensis*) and Wanjincheng (*C. sinensis*) types, as well as the transgenic Wanjincheng overexpressing CsBZIP40. Both the wild type and the transgenic plants were cultivated in a greenhouse at a temperature of 28°C. Cultures of XccYN1 were maintained at 28°C in a peptone-yeast extract-malt extract medium containing 1.5% (w/v) D-glucose [29], while XccYN1 was isolated in China’s Yunnan province, where it originated from infected naturally via sweet orange leaves [30].

### *In silico* analysis

The CsBZIP40, CsWRKY43, CsPrx53, and CsSOD13 sequences (Tables S1–S4, see online supplementary material) were accessed on the CPBD platform (http://citrus.hzau.edu.cn) [31, 32]. Mega X [33] was used to align protein sequences and construct phylogenetic trees. GSDS V2.0 (http://gsds.gao-lab.org) [34] was used to visualize gene structures. JASPAR V2020 (http://jaspar.genereg.net) [35] was adopted for the prediction of TF-binding sites. Meanwhile, HMMER (http://hmmer.org) [36] was used for the prediction of functional domains. Moreover, the Primer-BLAST (https://www.ncbi.nlm.nih.gov/tools/primer-blast) was used to construct the qRT-PCR primers, employing the *C. sinensis* mRNA database as a reference.

### RNAi and overexpression plasmids construction

The overexpression plasmids were made by amplification of the complete open reading frames (ORFs) of *CsBZIP40*, *CsWRKY43*, *CsPrx53*, and *CsSOD13* using the primer pairs listed below. These include the F_OEC-CsBZIP40_/R_OEC-CsBZIP40_, F_OEC-CsWRKY43_/R_OEC-CsWRKY43_, F_OEC-CsPrx53_/R_OEC-CsPrx53_ and F_OEC-CsSOD13_/R_OEC-CsSOD13_ (Table S5, see online supplementary material). The amplification products were then inserted into the pGLNe vector (Fig. S1A–**S1**D, see online supplementary material). To generate the RNAi vectors, a 304-bp fragment was amplified using the F_RIC-CsWRKY43_ and R_RIC-CsWRKY43_ primers and fused within the pUC-RANi vector. The final vector was made by inserting the RNAi sequence into pLGNe (Fig. S1E, see online supplementary material).

### RNA sequencing (RNA-seq) analysis

Wild-type (WT) and CsBZIP40-overexpressing Wanjincheng plants were used for RNA-seq. The leaves of the plants were harvested and an RNA miniprep kit (AidLab, China) was used for the total RNA extraction via Kit’s instructions. Subsequently, the samples were sent to Majorbio Inc. for RNA-seq on an Illumina Novaseq 6000 instrument (San Diego, California, USA) and analysed using the Majorbio cloud platform. After sequencing, clean data were mapped to the *C. sinensis* genome V1.0 in CPDB [31]. When analysing transcripts using the FPKM (fragments per kilobase of exon per million fragments mapped) method, genes were considered to be differentially expressed if they had a fold change |fold change (FC)| ≥ 1 and a false discovery rate (FDR) < 0.05. These genes were referred to as DEGs. Functional enrichment analysis was conducted using MapMan V3.6 based on the biotic stress pathway data in the included *C. sinensis* reference dataset.

### *Xcc* infection

Healthy leaves were freshly collected from Wanjincheng and Calamondin plants. They were injected with *Xcc* culture (OD_600_ = 0.5) and incubated at 28°C. Samples were collected from these leaves at 0, 6, 12, 24, 36, and 48 hours post-inoculation (hpi), for qRT-PCR analysis of *Xcc*.

### Transgenic plant development and validation

*Agrobacterium tumefaciens* were used for Wanjincheng shoot transformation, as reported previously [12, 26]. Histochemical analyses were conducted by performing β-glucuronidase (GUS) staining [26, 37]. The presence of the transgenes in CsBZIP40 and CsWRKY43 overexpression plants and CsWRKY43 RNAi plants was confirmed using the primer pairs F_OEID-CsBZIP40_/R_OEID-CsBZIP40_, F_OEID-CsWRKY43_/R_OEID-CsWRKY43_, and F_RID-CsWRKY43_/R_RID-CsWRKY43_, respectively (Table S5, see online supplementary material). Moreover, the qRT-PCR was further utilized to evaluate and confirm CsWRKY43 and CsBZIP40 expression in these generated transgenic plants.

### Evaluation of CBC resistance

*Xcc* cultures were applied to leaves that were six months old and were in healthy condition [2, 26, 38]. Briefly, 12 leaves from each plant were punctured using a 0.5-mm pin (24 punctures/leaf). After that, 1 μL of *Xcc* suspension containing 1.10^5^ CFU·mL^−1^ of liquid was administered into each puncture. Additionally, the cotton that had been soaked in double-distilled water (ddH_2_O) was used to provide treatment to the petioles. The severity of infections was then measured 10 days after infection (dpi). For quantifying CBC resistance, the disease index (DI) was selected [12, 38]. As previously described, *Xcc* infiltration experiments were also performed [12, 38, 44].

### Subcellular localization analysis

The CsWRKY43 subcellular localization was assessed by initially amplifying the ORFs corresponding to this gene without the corresponding stop codons and inserting them into the pBI221 vector with a GFP-encoding gene. The resultant fusion constructs or empty vector controls were inserted into *A. thaliana* protoplasts. At 48 hours post-infection (hpi), the GFP signal was evaluated in the cells via laser scanning confocal microscopy (LSM 510 Meta, Zeiss, Germany).

### Dual-luciferase reporter (dual-LUC) assay

Reporter constructs were developed by cloning the 1-kb promoters of *CsWRKY43*, *CsPrx53*, and *CsSOD13*. The pGreenII 0800 vector was then modified to include the desired promoters. The effector constructs were made by fusing the complete ORFs of CsBZIP40 and CsWRKY43 inside the pBI121-M vector, downstream of the CaMV35S promoter. Tobacco leaves were agroinfiltrated with *A. tumefaciens* (GV3101) carrying either the constructs or an empty vector control. The Dual-Glo® Luciferase Assay System (Promega, USA) was used to analyse Firefly and Renilla luminescence.

### Yeast one-hybrid (Y1H) assay

The pAbAi vector (bait) was cloned with the 1-kb promoters of CsWRKY43, CsPrx53, and CsSOD13. The ORFs of CsBZP40 and CsWRKY43 were also cloned into pGADT7 (prey). The yeast AH109 cells were transfected with the constructs using polyethylene glycol/lithium acetate [39]. Subsequently, we evaluated the transformants in SD media devoid of leucine (Leu), tryptophan (Trp), and histidine (His) (SD/−Leu/−Trp/-His) supplemented with Aureobasidin A (AbA) to determine the nature of the interactions between the components.

### Transcriptional activation activity assay

Transcriptional activity was analysed as reported in a previous paper [40]. In short, the whole open reading frame (ORF) for *CsWRKY43* was cloned into the pGBKT7 vector (Clontech, USA) immediately after the GAL4 DNA-binding domain (GAL4BD). The resulting vector was used to convert Y2HGold yeast cells, which were plated on a synthetic dropout medium (SD) devoid of tryptophan and leucine (SD/−Leu/−Trp). Cultures of transformed cells were made in synthetic dropout medium (SD) lacking tryptophan and histidine (SD/−Leu/−Trp/–His) or lacking tryptophan, histidine, and adenine (SD/−Leu/−Trp/-His/−Ade) with or without X-α-gal (5-bromo-4-chloro-3-indolyl-α-D-galactoside) (containing 0 or 40 μg·mL^−1^) to analyse the transcriptional activation of CsWRKY43.

### Electrophoretic mobility shift assay (EMSA)

Cloning of CsBZIP40 and CsWRKY43 ORFs into a pGEX-4 T-1 vector (GST tag-containing) allowed for the proteins to be produced in *Escherichia coli* BL21 cells after the stop codons were removed. The proteins were expressed and purified as described in an earlier study [41]. TF-binding sites (TFBS) in the *CsWRKY43*, *CsPrx53*, and *CsSOD13* promoter regions were predicted using JASPAR V2020 (http://jaspar.genereg.net) [35]. The wild-type (WT) probe *CsWRKY43*, the mutant type (MT) probe *CsWRKY43*, the wild-type (WT) probe *CsPrx53*, and the mutated type (MT) probe *CsSOD13* were used to design biotin-labeled single-stranded oligonucleotides containing the TFBS (Sangon Biotechnology, China). The LightShift Chemiluminescent EMSA Kit (Thermo Scientific, USA) was used to perform the assays according to the manufacturer’s instructions.

### Transient transformation of citrus

Overexpression vectors for CsWRKY43, CsPrx53, and CsSOD13 were introduced into *A. tumefaciens* (EHA105). After inoculating citrus leaves with *A. tumefaciens*, the cultures were kept at 28°C for five days before being sampled. Eventually, the CsWRKY43, CsPrx53, and CsSOD13 expression levels were evaluated using qRT-PCR.

### Virus-induced gene silencing (VIGS)

Primer pairs F_VIGS-CsPrx53_/R_VIGS-CsPrx53_ and F_VIGS-CsSOD13_/R_VIGS-CsSOD13_ were used for the amplification of VIGS fragments (Table S5, see online supplementary material), which were then integrated into the TRV2 vector to construct TRV2-CsPrx53 and TRV2-CsSOD13. VIGS transformation following *A. tumefaciens* infiltration was conducted as described earlier [42]. After 30 days, plants showing green fluorescence under UV light were sampled for further analysis. The primers used for TRV1 detection were F_DEC-TRV1_/R_DEC-TRV1_, and the primers used for TRV2 detection were F_DEC-TRV2_/R_DEC-TRV2_ (Table S5, see online supplementary material). qRT-PCR was used to test the silencing efficiency [42].

### qRT-PCR

Liquid nitrogen was used to smash frozen samples of leaves. After that, total RNA was extracted with the use of a commercial RNA extraction kit from AidLab (China) and reverse transcribed with a commercial kit (TaKaRa, Japan). The obtained cDNA was put through a qRT-PCR analysis utilizing a Quantagene Real-Time System q225 (Novogene, China) and a SYBR Premix kit (Bio-Rad, USA). The following served as the procedures: a pre-denaturation period of 5 minutes at 95°C is followed by 40 cycles of denaturation for 10 seconds at 95°C and annealing and extension for 30 seconds at 56°C. Each 12-uL reaction included 6 uL of SYBR Green PCR mix, 0.5 uL of primer, and 100 ng of cDNA. The primer pairs F_RT-CsBZIP40_/R_RT-CsBZIP40_, F_RT-CsWRKY43_/R_RT-CsWRKY43_, F_RT-CsPrx53_/R_RT-CsPrx53_, and F_RT-CsSOD13_/R_RT-CsSOD13_ (Table S5, see online supplementary material) were utilized for these assays. The 2^-∆∆CT^ approach was used to establish relative gene expression [43].

### Biochemical assay

Following the manufacturer’s instructions, we evaluated the levels of hydrogen peroxide (H_2_O_2_) and superoxide radicals (O_2_^−^), superoxide dismutase (SOD) and peroxidase (POD) using SinoBestBio kits (Shanghai, China). The citrus leaves were finely powdered using a mortar and pestle. Then, 900 mL of ice-cold physiological saline solution was immediately mixed with 100 mg of the frozen powder. The supernatant was collected after centrifuging the homogenate at a rate of 1000 r·min^−1^ at 20°C. The ROS concentrations and enzyme activity were measured in the supernatant (500 mL). The samples were run through the lab a total of three times.

### Statistical analysis

The results were summarized by using means ± standard deviations (SDs), respectively. Prism V8 (GraphPad, USA) was used throughout every stage of the data analysis process. Comparisons of the data were carried out using either Duncan’s multiple range test for analysis of variance (ANOVA) or two-tailed *t*-tests.

## Supplementary Material

Web_Material_uhad138

## Data Availability

The raw RNA-Seq data are archived as Sequence Read Archive (SRA) in the National Center for Biotechnology Information (NCBI) with an accession number **PRJNA909460**. Other data supporting the findings of this study are available within the article and supplementary data.

## References

[1] Reboledo G , Agorio A, Ponce De León I. Moss transcription factors regulating development and defense responses to stress. J Exp Bot. 2022;73:4546–61 35167679 10.1093/jxb/erac055

[2] Li Q , Jia R, Dou W et al. CsBZIP40, a BZIP transcription factor in sweet orange, plays a positive regulatory role in citrus bacterial canker response and tolerance. PLoS One. 2019;14:e0223498 31584990 10.1371/journal.pone.0223498 PMC6777757

[3] Barah P , Jayavelu ND, Mundy J et al. Genome scale transcriptional response diversity among ten ecotypes of *Arabidopsis thaliana* during heat stress. Front Plant Sci. 2013;4:532 24409190 10.3389/fpls.2013.00532 PMC3872818

[4] Jin Z , Xu W, Liu A. Genomic surveys and expression analysis of bZIP gene family in castor bean (*Ricinus communis* L.). Planta. 2014;239:299–312 24165825 10.1007/s00425-013-1979-9

[5] Nijhawan A , Jain M, Tyagi AK et al. Genomic survey and gene expression analysis of the basic leucine zipper transcription factor family in rice. Plant Physiol. 2008;146:333–50 18065552 10.1104/pp.107.112821 PMC2245831

[6] Zhang M , Liu Y, Li Z et al. The bZIP transcription factor GmbZIP15 facilitates resistance against. iScience. 2021;24:102642 34151234 10.1016/j.isci.2021.102642 PMC8188564

[7] He Q , Cai H, Bai M et al. A soybean bZIP transcription factor gene *GmbZIP2* confers drought and salt resistances in transgenic plants. Int J Mol Sci. 2020;21:670 31968543 10.3390/ijms21020670 PMC7013997

[8] Lim CW , Baek W, Lim S et al. Expression and functional roles of the pepper pathogen-induced bZIP transcription factor CabZIP2 in enhanced disease resistance to bacterial pathogen infection. Mol Plant-Microbe Interact. 2015;28:825–33 25738319 10.1094/MPMI-10-14-0313-R

[9] Li Q , Yu H, Cao PB et al. Explosive tandem and segmental duplications of multigenic families in *Eucalyptus grandis*. Genome Biol Evol. 2015;7:1068–81 25769696 10.1093/gbe/evv048 PMC4419795

[10] Mittler R , Zandalinas SI, Fichman Y et al. Reactive oxygen species signalling in plant stress responses. Nat Rev Mol Cell Biol. 2022;23:663–79 35760900 10.1038/s41580-022-00499-2

[11] Hirt H . Aquaporins link ROS signaling to plant immunity. Plant Physiol. 2016;171:1540 27385821 10.1104/pp.16.00433 PMC4936576

[12] Li Q , Hu AH, Qi JJ et al. CsWAKL08, a pathogen-induced wall-associated receptor-like kinase in sweet orange, confers resistance to citrus bacterial canker via ROS control and JA signaling. Hortic Res. 2020;7:15 32257228 10.1038/s41438-020-0263-y PMC7109087

[13] Mittler R , Blumwald E. The roles of ROS and ABA in systemic acquired acclimation. Plant Cell. 2015;27:64–70 25604442 10.1105/tpc.114.133090 PMC4330577

[14] Alves MS , Dadalto SP, Gonçalves AB et al. Plant bZIP transcription factors responsive to pathogens: a review. Int J Mol Sci. 2013;14:7815–28 23574941 10.3390/ijms14047815 PMC3645718

[15] Amorim LLB , da Fonseca Dos Santos R, Neto JPB et al. Transcription factors involved in plant resistance to pathogens. Curr Protein Pept Sci. 2017;18:335–51 27323805 10.2174/1389203717666160619185308

[16] Kaminaka H , Näke C, Epple P et al. bZIP10-LSD1 antagonism modulates basal defense and cell death in Arabidopsis following infection. EMBO J. 2006;25:4400–11 16957775 10.1038/sj.emboj.7601312 PMC1570446

[17] Li Q , Qi J, Qin X et al. Systematic identification of lysin-motif receptor-like kinases (LYKs) in *Citrus sinensis*, and analysis of their inducible involvements in citrus bacterial canker and phytohormone signaling. Sci Hortic. 2021;276:109755

[18] Fawal N , Li Q, Savelli B et al. PeroxiBase: a database for large-scale evolutionary analysis of peroxidases. Nucleic Acids Res. 2013;41:D441–4 23180785 10.1093/nar/gks1083 PMC3531118

[19] Pandey S , Fartyal D, Agarwal A et al. Abiotic stress tolerance in plants: myriad roles of ascorbate peroxidase. Front Plant Sci. 2017;8:581 28473838 10.3389/fpls.2017.00581 PMC5397514

[20] Smirnoff N , Arnaud D. Hydrogen peroxide metabolism and functions in plants. New Phytol. 2019;221:1197–214 30222198 10.1111/nph.15488

[21] Tian S , Wang X, Li P et al. Plant aquaporin AtPIP1;4 links apoplastic H_2_O_2_ induction to disease immunity pathways. Plant Physiol. 2016;171:1635–50 26945050 10.1104/pp.15.01237 PMC4936539

[22] El-Shetehy M , Wang C, Shine MB et al. Nitric oxide and reactive oxygen species are required for systemic acquired resistance in plants. Plant Signal Behav. 2015;10:e998544 26375184 10.1080/15592324.2014.998544 PMC4883869

[23] Foyer CH . How plant cells sense the outside world through hydrogen peroxide. Nature. 2020;578:518–9 32094917 10.1038/d41586-020-00403-y

[24] Planas-Riverola A , Markaide E, Caño-Delgado AI. New role for LRR-receptor kinase in sensing of reactive oxygen species. Trends Plant Sci. 2021;26:102–4 33309457 10.1016/j.tplants.2020.11.011

[25] Wu F , Chi Y, Jiang Z et al. Hydrogen peroxide sensor HPCA1 is an LRR receptor kinase in Arabidopsis. Nature. 2020;578:577–81 32076270 10.1038/s41586-020-2032-3

[26] He Y , Jia R, Qi J et al. Functional analysis of citrus AP2 transcription factors identified CsAP2-09 involved in citrus canker disease response and tolerance. Gene. 2019;707:178–88 30991097 10.1016/j.gene.2019.04.021

[27] Khan M , Hu J, Dahro B et al. ERF108 from *Poncirus trifoliata* (L.) Raf. Functions in cold tolerance by modulating raffinose synthesis through transcriptional regulation of PtrRafS. Plant J. 2021;108:705–24 34398993 10.1111/tpj.15465

[28] Jiang Y , Tong S, Chen N et al. The PalWRKY77 transcription factor negatively regulates salt tolerance and abscisic acid signaling in Populus. Plant J. 2021;105:1258–73 33264467 10.1111/tpj.15109

[29] Hu Y , Zhang J, Jia H et al. Lateral organ boundaries 1 is a disease susceptibility gene for citrus bacterial canker disease. Proc Natl Acad Sci U S A. 2014;111:E521–9 24474801 10.1073/pnas.1313271111 PMC3910620

[30] Li Q , Qin X, Qi J et al. CsPrx25, a class III peroxidase in *Citrus sinensis*, confers resistance to citrus bacterial canker through the maintenance of ROS homeostasis and cell wall lignification. Hortic Res. 2020;7:192 33328465 10.1038/s41438-020-00415-9 PMC7705758

[31] Liu H , Wang X, Liu S et al. Citrus pan-genome to breeding database (CPBD): a comprehensive genome database for citrus breeding. Mol Plant. 2022;15:1503–5 36004795 10.1016/j.molp.2022.08.006

[32] Wang J , Chen D, Lei Y et al. *Citrus sinensis* annotation project (CAP): a comprehensive database for sweet orange genome. PLoS One. 2014;9:e87723 24489955 10.1371/journal.pone.0087723 PMC3905029

[33] Kumar S , Stecher G, Li M et al. MEGA X: molecular evolutionary genetics analysis across computing platforms. Mol Biol Evol. 2018;35:1547–9 29722887 10.1093/molbev/msy096 PMC5967553

[34] Hu B , Jin J, Guo AY et al. GSDS 2.0: an upgraded gene feature visualization server. Bioinformatics. 2015;31:1296–7 25504850 10.1093/bioinformatics/btu817 PMC4393523

[35] Fornes O , Castro-Mondragon JA, Khan A et al. JASPAR 2020: update of the open-access database of transcription factor binding profiles. Nucleic Acids Res. 2020;48:D87–92 31701148 10.1093/nar/gkz1001 PMC7145627

[36] Finn RD , Clements J, Eddy SR. HMMER web server: interactive sequence similarity searching. Nucleic Acids Res. 2011;39:W29–37 21593126 10.1093/nar/gkr367 PMC3125773

[37] Sendín LN , Orce IG, Gómez RL et al. Inducible expression of Bs2 R gene from Capsicum chacoense in sweet orange (*Citrus sinensis* L. Osbeck) confers enhanced resistance to citrus canker disease. Plant Mol Biol. 2017;93:607–21 28155188 10.1007/s11103-017-0586-8

[38] Peng A , Chen S, Lei T et al. Engineering canker-resistant plants through CRISPR/Cas9-targeted editing of the susceptibility gene CsLOB1 promoter in citrus. Plant Biotechnol J. 2017;15:1509–19 28371200 10.1111/pbi.12733 PMC5698050

[39] Gietz RD , Schiestl RH. Quick and easy yeast transformation using the LiAc/SS carrier DNA/PEG method. Nat Protoc. 2007;2:35–7 17401335 10.1038/nprot.2007.14

[40] Long Q , Du MX, Long JH et al. Transcription factor WRKY22 regulates canker susceptibility in sweet orange (*Citrus sinensis* Osbeck) by enhancing cell enlargement and CsLOB1 expression. Hortic Res. 2021;8:15 33642585 10.1038/s41438-021-00486-2 PMC7917094

[41] Duan S , Jia H, Pang Z et al. Functional characterization of the citrus canker susceptibility gene *CsLOB1*. Mol Plant Pathol. 2018;19:1908–16 29461671 10.1111/mpp.12667 PMC6638005

[42] Wang F , Wang M, Liu X et al. Identification of putative genes involved in limonoids biosynthesis in citrus by comparative transcriptomic analysis. Front Plant Sci. 2017;8:782 28553308 10.3389/fpls.2017.00782 PMC5427120

[43] Livak KJ , Schmittgen TD. Analysis of relative gene expression data using real-time quantitative PCR and the 2−ΔΔCT method. Methods. 2001;25:402–8 11846609 10.1006/meth.2001.1262

[44] Fu J , Yu Q, Zhang C et al. CsAP2-09 confers resistance against citrus bacterial canker by regulating CsGH3.1L-mediated phytohormone biosynthesis. Int J Biol Macromol. 2023;229:964–73 36587648 10.1016/j.ijbiomac.2022.12.311

