# Supplementary material for: CsBZIP40 confers resistance against citrus bacterial canker by repressing CsWRKY43-CsPrx53/CsSOD13 cascade mediated ROS scavenging
Source: Hortic Res. 2023 Jul 11;10(8):uhad138. doi: 10.1093/hr/uhad138 (PMC10421728; doi:10.1093/hr/uhad138)

**Supplementary Figures**

**Figure S1: Schematic overview of the expression vectors used for citrus transformation.** **(A)** CsBZIP40 overexpression vector. **(B)** CsWRKY43 overexpression vector. **(C)** CsPrx53 overexpression vector. **(D)** CsSOD13 overexpression vector. **(E)** CsWRKY43 RNAi vector. P_35S_, cauliflower mosaic virus 35S promoter; T_NOS_, NOS terminator; GUS: β-glucuronidase; NPTII: β-glucuronidase and NPT integrate coding genes; LB: left border; RB: right border; FLAG: FLAG tag.


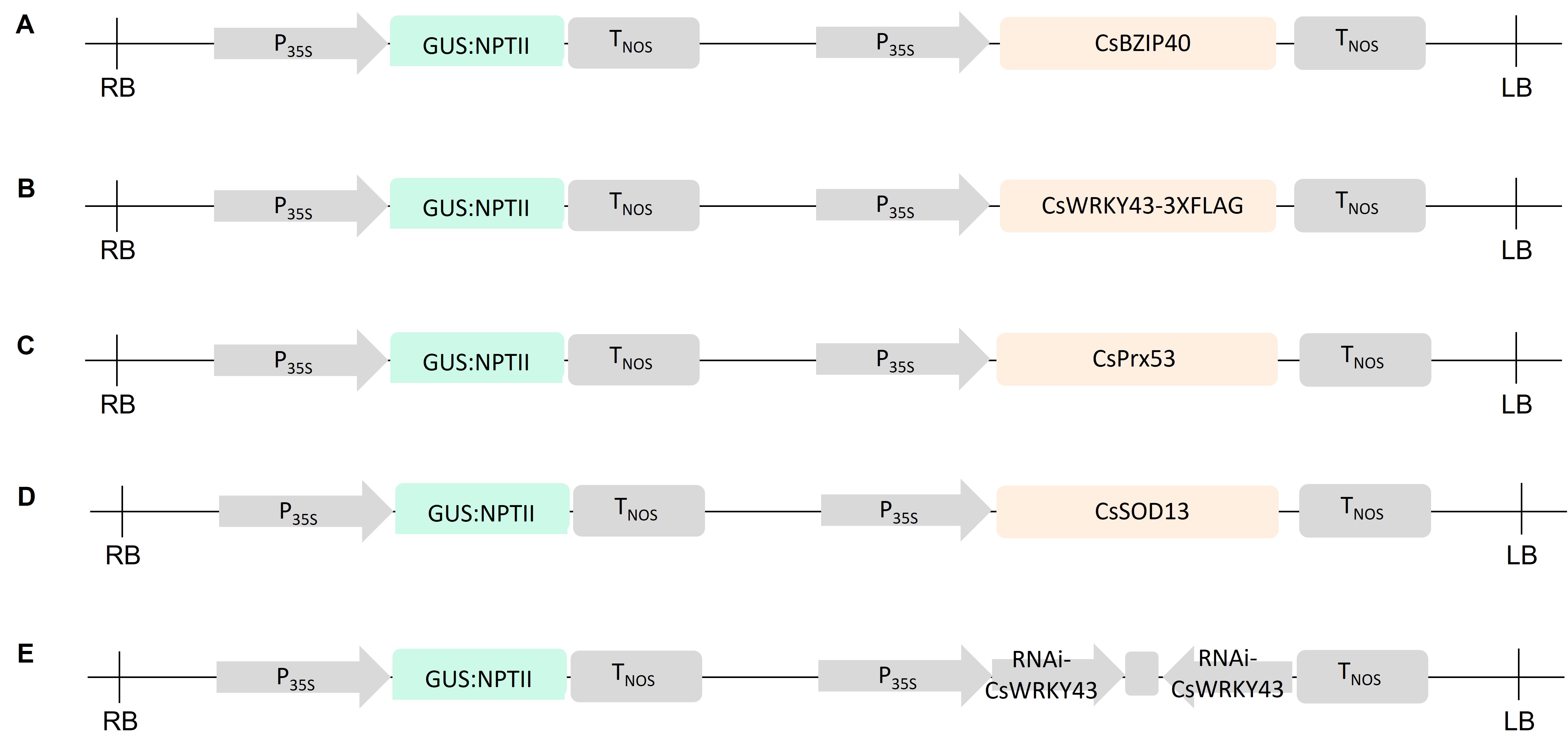


**Figure S2. Identification of transgenic plants overexpressing CsBZIP40. (A)** GUS staining of transgenic plants. Scale bar = 7 mm. **(B)** PCR-based validation of generated transgenic plants.


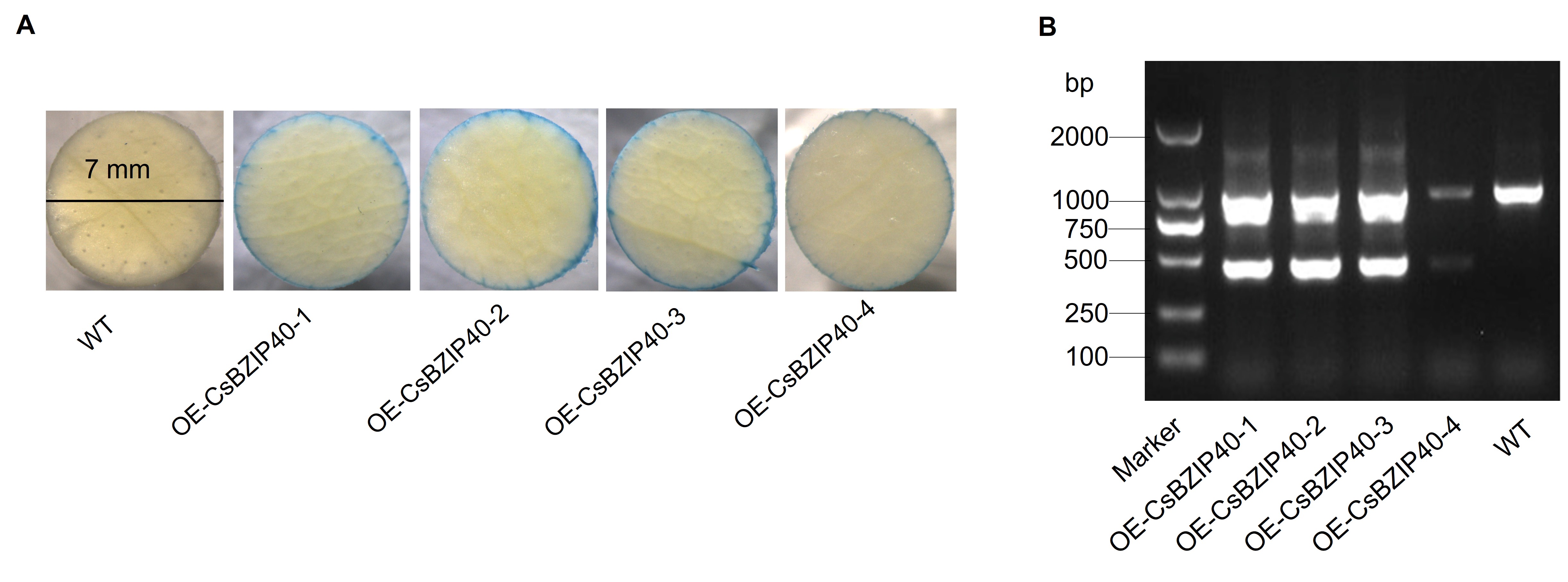


**Figure S3: CsBZIP40-binding elements in the *CsWRKY43* promoter. (A)** Predicted binding site positions within the 1000-bp region upstream to the start codon for *CsWRKY43* promoter. **(B)** Consensus sequence logo corresponding to the predicted CsBZIP40 binding site. **(C)** Relative scores for predicted CsBZIP40 binding sites. JASPAR V2020 was used for all predictions using an 85.0% relative score threshold, with the homologous *A. thaliana* transcription factor gene *AtTGA9* (TAIR ID: *AT1G08320.1*) serving as the query.


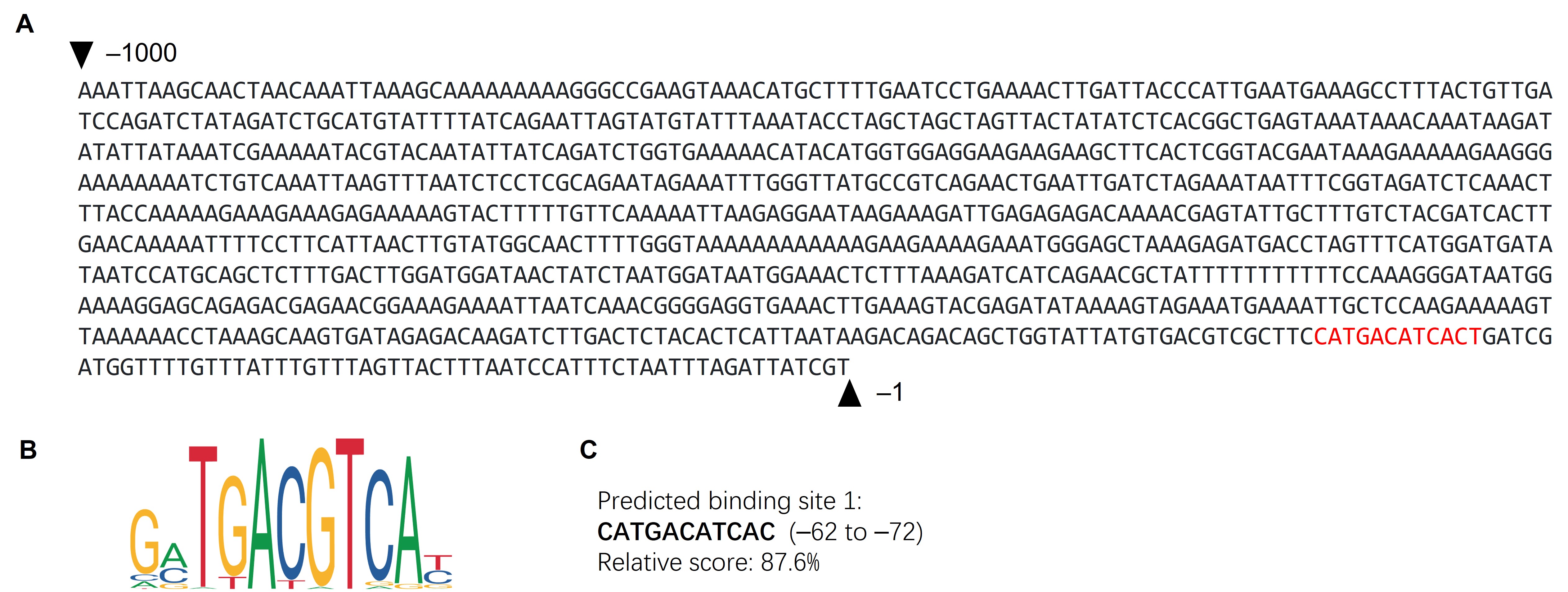


**Figure S4: Self-activation of the *CsWRKY43* promoter and inhibition in the presence of AbA.**


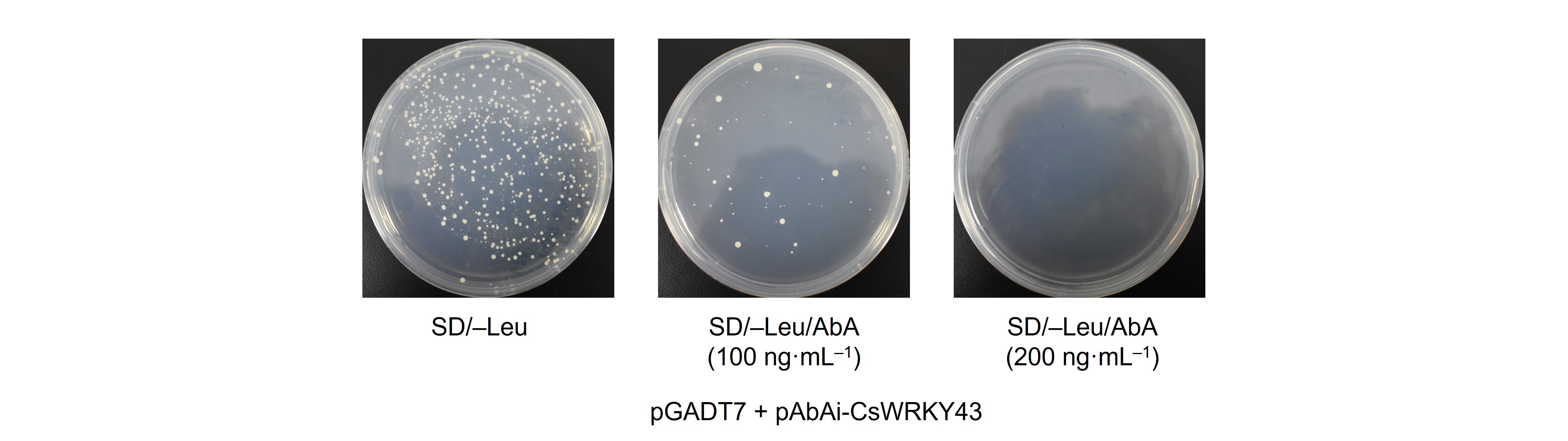


**Figure S5: Identification and phenotypic characterization of transgenic plants. (A)** GUS staining of transgenic plants. Scale bar = 7 mm. **(B)** PCR-based validation of generated transgenic plants. M: DNA ladder. **(C)** Phenotypic assessment of transgenic plants overexpressing CsWRKY43. Scale bar = 30 cm. WT: wild type Wanjincheng; OE-CsWRKY43-1, 2 and 3: transgenic Wanjincheng overexpressing CsWRKY43. Ri-CsWRKY43-1, 2 and 3: transgenic Wanjincheng repressing CsWRKY43.


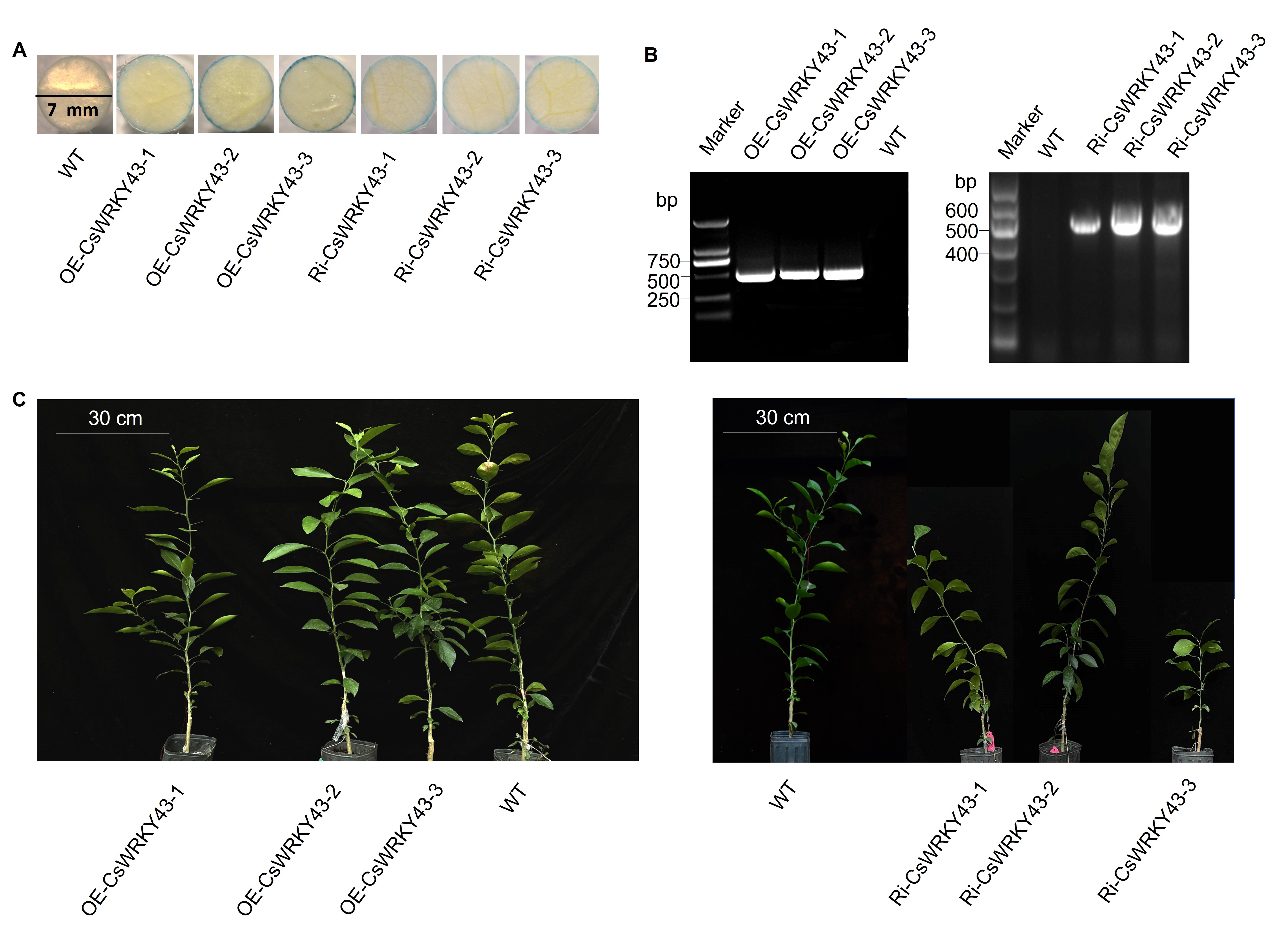


**Figure S6: CsWRKY43-binding elements in the *CsPrx53* promoter. (A)** Predicted binding site positions within the 1000-bp region upstream to the start codon for *CsPrx53* promoter. **(B)** Consensus sequence logo corresponding to the predicted CsWRKY43 binding site. **(C)** Relative scores for predicted CsWRKY43 binding sites. JASPAR V2020 was used for all predictions using an 85.0% relative score threshold, with the homologous *A. thaliana* transcription factor gene *AtWRKY57* (TAIR ID: *AT1G69310.1*) serving as the query.


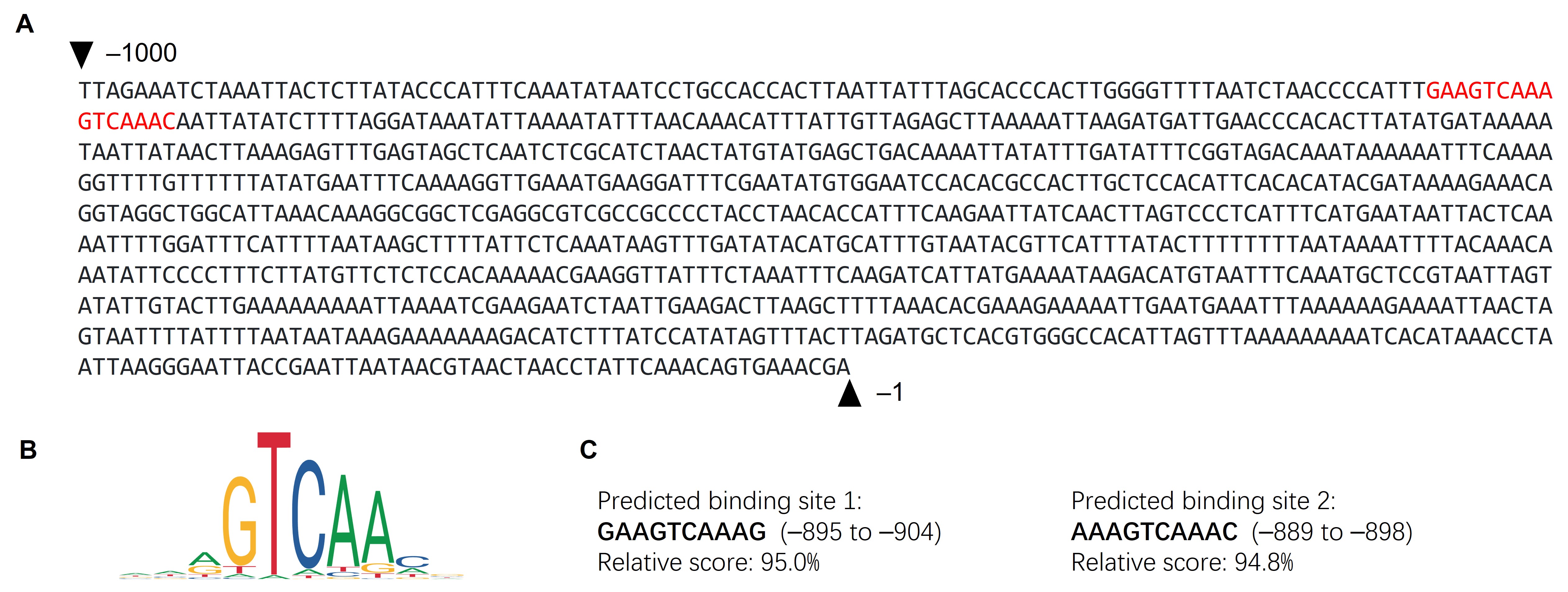


**Figure S7: CsWRKY43 binding elements in the *CsSOD13* promoter. (A)** Predicted binding site positions within the 1000-bp region upstream to the start codon for *CsSOD13* promoter. **(B)** Consensus sequence logo corresponding to the predicted CsWRKY43 binding site. **(C)** Relative scores for predicted CsWRKY43 binding sites. JASPAR V2020 was used for all predictions using an 85.0% relative score threshold, with the homologous *A. thaliana* transcription factor gene *AtWRKY57* (TAIR ID: *AT1G69310.1*) serving as the query.


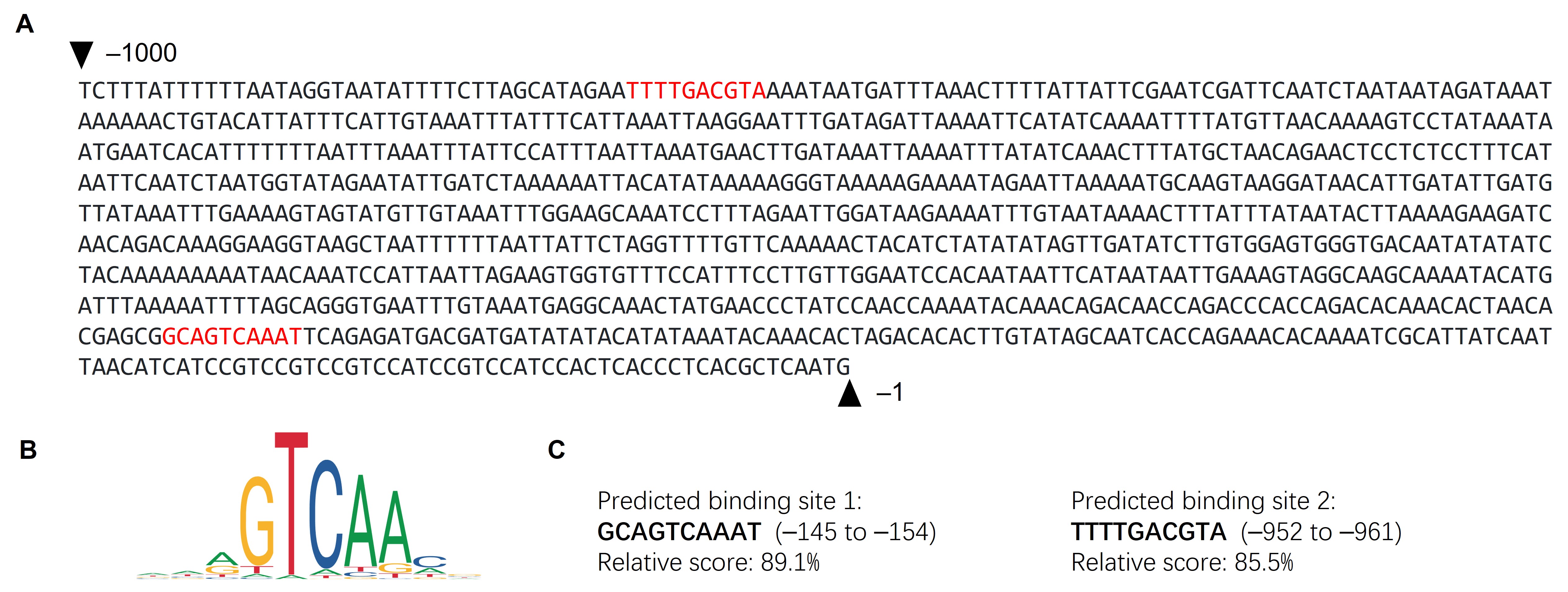


**Figure S8: Self-activation of promoters and inhibition in the presence of AbA. (A)** *CsPrx53* promoter. **(B)** *CsSOD13* promoter.


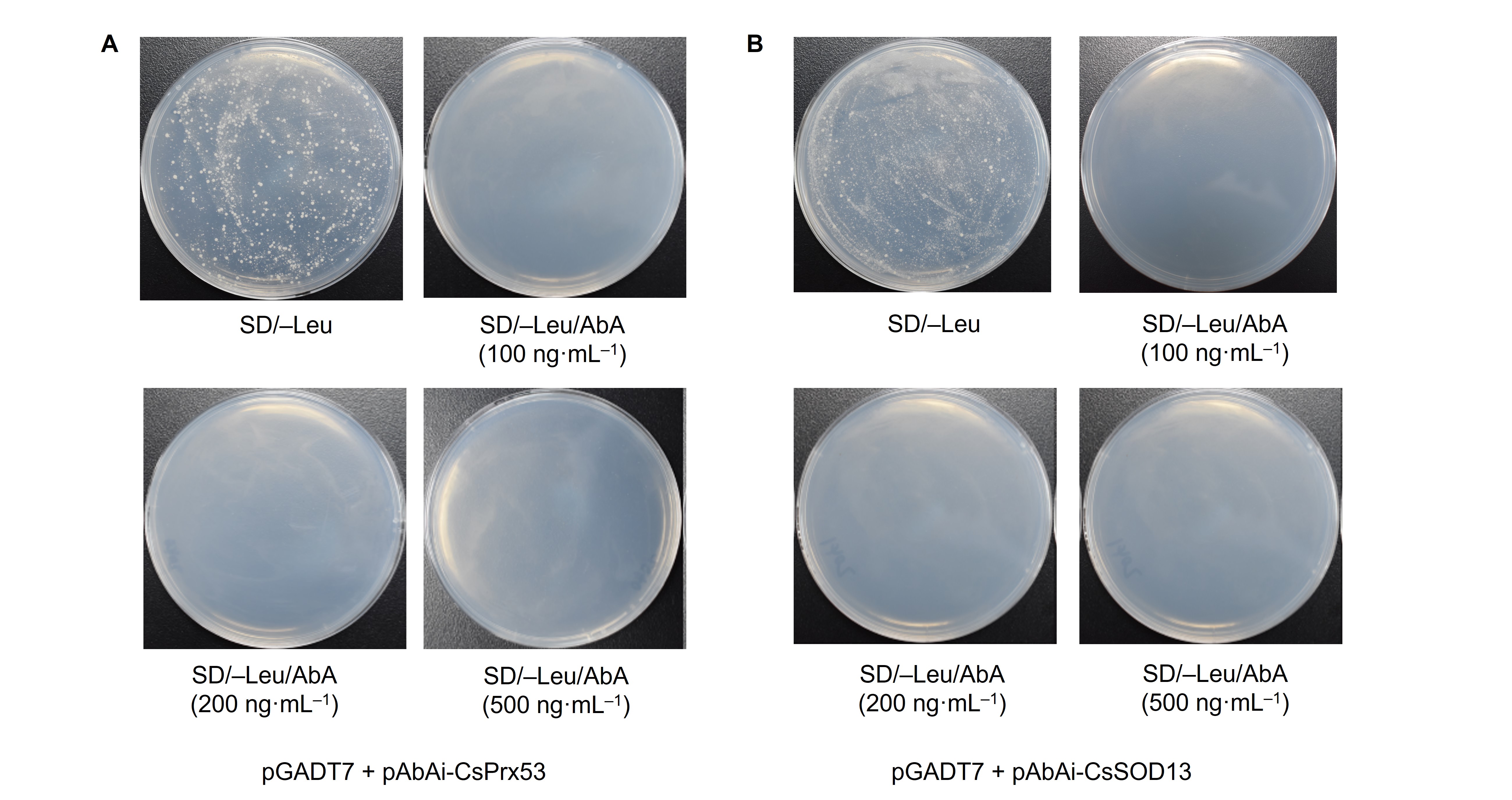


**Figure S9: Bioinformatics characterization of CsPrx53.** Chromosomal positioning was determined using CPDB. The gene structure was visualized with GSDS V2.0. The functional domains and signal peptide were analyzed with HMMER. bp: base pair; aa: amino acid.


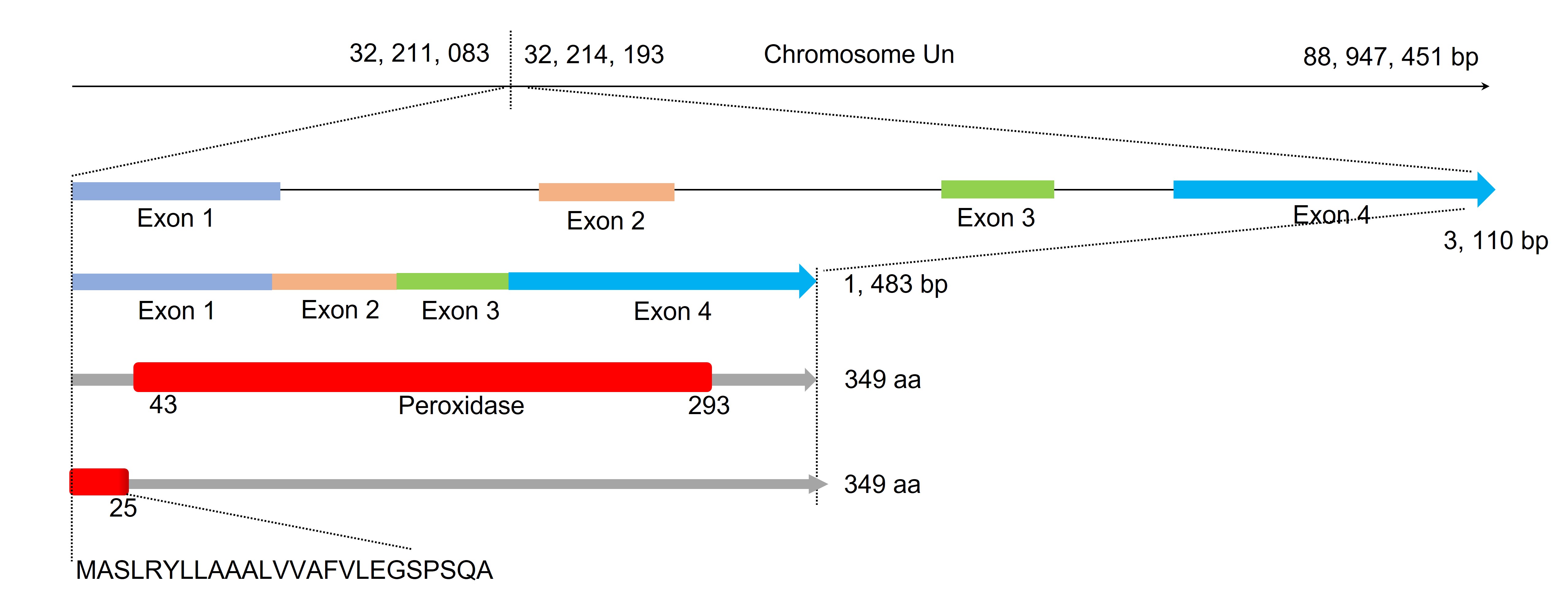


**Figure S10: Bioinformatics characterization of CsSOD13.** Chromosomal positioning was determined using CPDB. The gene structure was visualized with GSDS V2.0. The functional domains and signal peptide were analyzed with HMMER. bp: base pair; aa: amino acid.


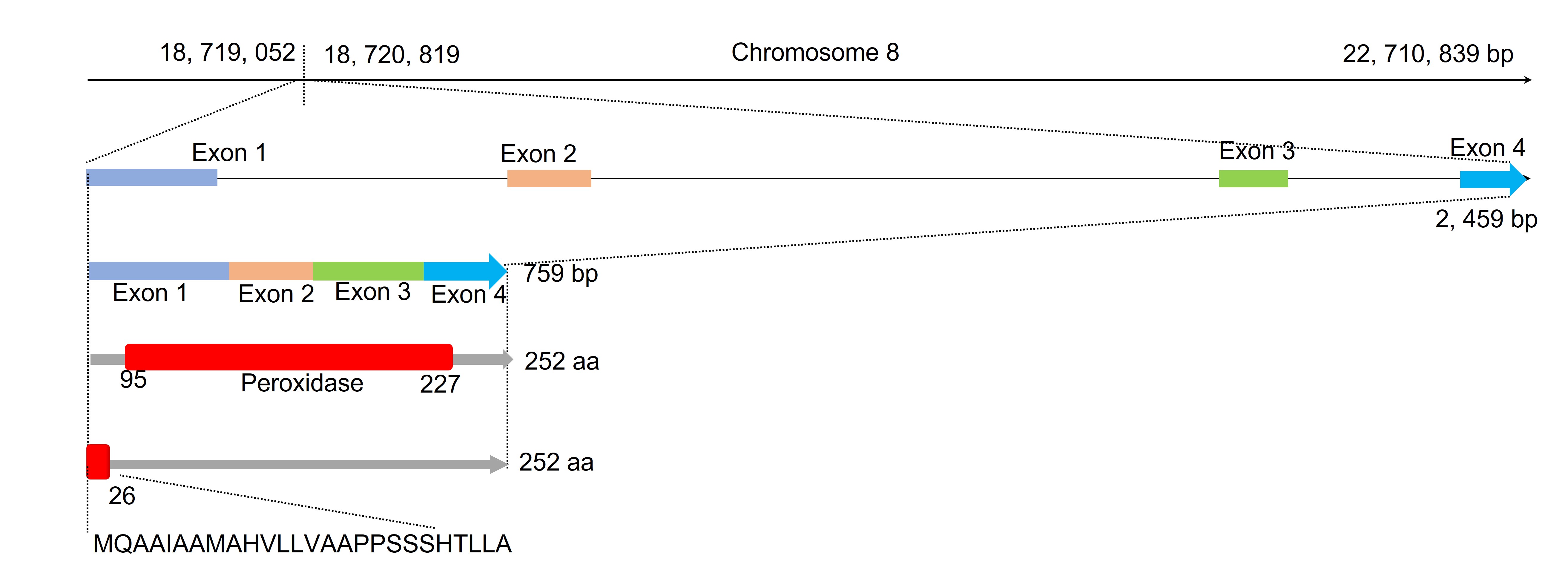


**Figure S11: CsPrx53 and CsSOD13 expression in Wanjincheng and Calamondin leaves following *Xcc* infection.** **(A)** CsPrx53 expression. **(B)** CsSOD13 expression. The expression was assessed using qRT-PCR, with CsActin as the normalization control. Data were compared using ANOVA and Duncan’s multiple range test.


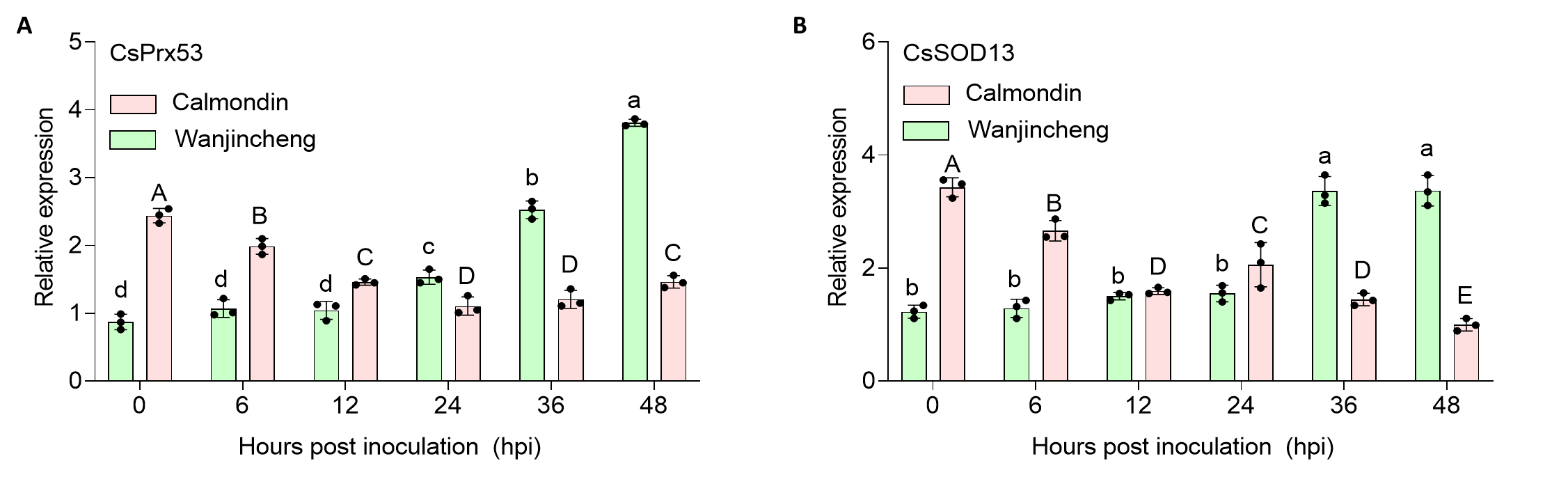


**Figure S12: Schemes of plasmids used for VIGS.** 35S, cauliflower mosaic virus 35S promoter; NOS, NOS terminator; GFP, green fluorescence protein; RdRp: RNA-dependent DNA polymerase; CP: coat protein; LB: left border; RB: right border.


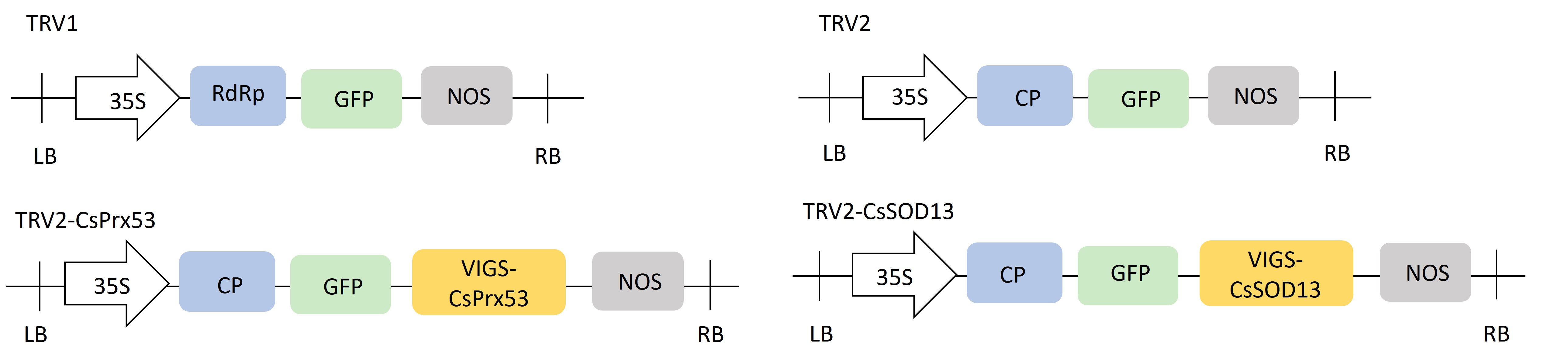


**Figure S13: Verification of VIGS plants using PCR.** **(A)** Verification of CsPrx53 VIGS plants. **(B)** Verification of CsSOD13 VIGS plants. In (A) – (B), M: DNA ladder. –: ddH_2_O as the negative control; +; plasmid as the positive control.


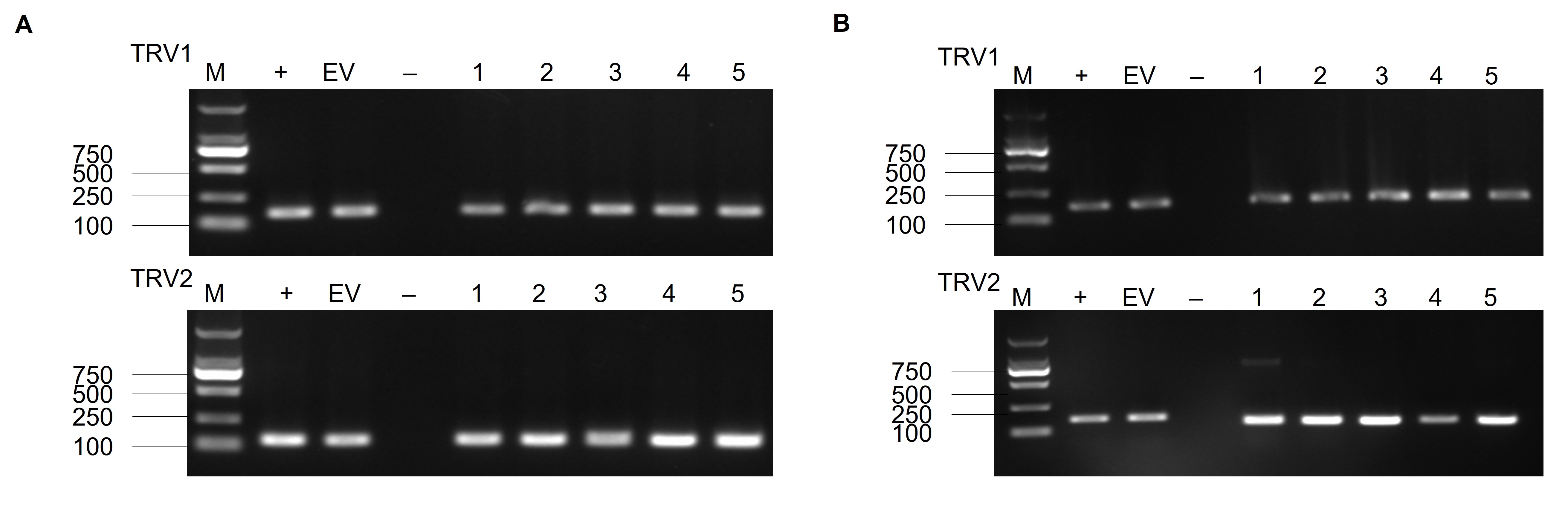


**Figure S14: Relative expression of CsWRKY43, CsPrx53 and CsSOD1 3 in transgenic plants repressing CsBZIP40. (A)** CsWRKY43 expression. **(B)** CsPrx53 expression.  **(C)** CsSOD13 expression. The assays were assessed using qRT-PCR, with CsActin serving as the normalization control. WT: wild type Wanjincheng; RNAi-CsBZIP40-1 and 2: transgenic Wanjincheng repressing CsBZIP40. Leaves were picked for all assays.


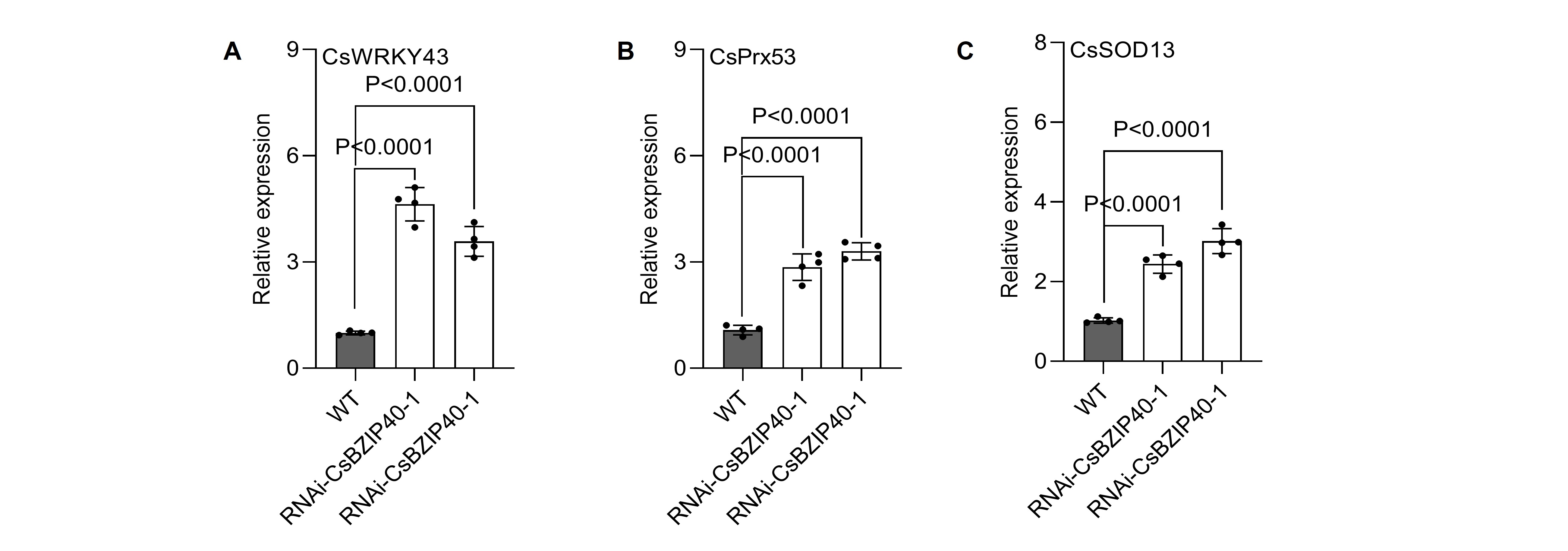

Supplement: Web_Material_uhad138 [file web_material_uhad138.zip › Supplementary Figures.docx]
